# How do people in prison access palliative care? A scoping review of models of palliative care delivery for people in prison in high-income countries

**DOI:** 10.1177/02692163241242647

**Published:** 2024-04-16

**Authors:** Emma Gilbert, Nick De Viggiani, Joana de Sousa Martins, Tanuka Palit, Jessica Sears, Daniel Knights, Audrey Roulston, Mary Turner, Lucy E Selman

**Affiliations:** 1 Palliative and End of Life Care Research Group, Bristol Medical School, University of Bristol, Bristol UK; 2 School of Health and Social Wellbeing, Unversity of the West of England, UK; 3 Academic Clinical Fellow Centre of Academic Primary Care, Bristol Medical School, University of Bristol, UK; 4 King’s College London, London, UK; 5 University of Cambridge, Cambridge, UK; 6 Professor of Social Work in Palliative Care, School of Social Sciences, Education and Social Work Queen’s University, Belfast, UK; 7 Reader in Health Services Research, University of Huddersfield, Huddersfield, UK

**Keywords:** Palliative care, hospice, hospice care, prisons, jails, scoping review, end of life care

## Abstract

**Background:**

An ageing prison population with complex health needs combined with punitive sentencing practices means palliative care for incarcerated individuals is increasingly important. However, there is limited evidence regarding the models of care delivery in high-income countries, and their associated challenges and benefits.

**Aim:**

To develop a typology of models of palliative care provision for incarcerated individuals, synthesise evidence of their outcomes and describe facilitators of and challenges in delivering different models of palliative and end-of-life care in prisons.

**Design:**

Scoping review following Arksey and O’Malley, with narrative synthesis. The protocol was registered prospectively (reviewregistry1260).

**Data sources:**

MEDLINE, EMBASE, CINAHL, PsycINFO, the Social Sciences Citation Index and grey literature were searched on 15th March 2023. The Mixed Methods Appraisal Tool (MMAT) was used for quality appraisal.

**Results:**

A total of 16,865 records were screened; 22 peer-reviewed articles and 18 grey literature sources met the inclusion criteria. Three models were identified: Embedded Hospice, Outsourcing Care and Community Collaboration. The Embedded Hospice model shows potential benefits for patients and prisons. Outsourcing Care may miss opportunities for comprehensive care. Collaborative Care relies on proactive prison-community relationships that could be formalised for improvement. Psychosocial and bereavement needs of those dying in prison and their caregivers lack sufficient documentation.

**Conclusion:**

Further research is needed to evaluate prison hospice costs and examine how prison hospices impact compassionate release usage. Beyond the USA, policies might formalise care pathways and recognise best practices. Further investigation to address psychosocial needs of people in prison with life-limiting illnesses and post-death bereavement support is required.

**What is already known about the topic?**

The ageing prison population is growing worldwide.
Access to quality palliative care for people in prison is a pressing requirement.
There are significant variations in how palliative care is delivered for people in prison across high-income countries.

**What this paper adds?**

We identified a typology of three models of care delivery for people in prison in high-income countries: (1) Embedded hospice model, typified by an interdisciplinary team and volunteer caregivers providing care on-site; (2) Outsourcing Care model, in which end-of-life care is provided outside the prison; (3) Collaborative community model, which involves prisons engagement with other healthcare facilities or practitioners.
Embedded hospice models in the USA are prevalent and demonstrate promising evidence for enhancing the care experience for recipients and peer caregivers. Chaplains, social workers and peer caregivers provide psychosocial support, yet documented assessment and strategies for managing the distinctive needs of this group and their families are lacking, despite their acknowledged complexity.

**Implications for practice, theory or policy**

Local policies should formalise pathways between community palliative care providers and prisons, emphasise flexible visiting and consider expanding the definition of ‘family’ to include fellow individuals in prison.
Prioritising consultations with stakeholders at the national governmental level on sentencing practices for older people in prison is essential.
Frameworks such as the UK’s Dying Well in Custody Charter can support national quality standards but require robust implementation and monitoring.

## Introduction

Prison populations in many high-income countries are growing, due to population ageing and increasingly punitive sentencing policies.^1,2^ Dedicated palliative care provision in prisons has become a pressing requirement; however, marked variability in models of care between high-income countries hinders meaningful comparison and the identification and implementation of best practice.^1^

People in prison deserve equivalent access to healthcare as the general population, and barriers to care delivery can be considered a human rights issue.^2,3^ Initial studies on palliative care in prisons worldwide highlight gaps in current provision and a lack of research evidence to inform practice.^3–7^ Services have developed at vastly different rates, both between high-income countries and within.^1,8^ Inequitable provisions for people in prison with life-limiting illnesses, who are often older, with multimorbidity and frailty, coupled with stringent early-release policies, continue to prevail.^1^ Countries differ significantly in compassionate early-release policies. Common elements considered for granting early release include offence type, reoffending risk and suitability for transfer to a non-prison setting. In France, a person is temporarily moved to hospital but will return if they recover, whereas in the U.S.A., compassionate release allows those in prison to seek early release for severe health issues including age-related conditions, with medical parole employed as the primary mechanism across states.^1^ Advocacy for compassionate release is in tension with a prevailing punitive approach that limits access to end-of-life care. This, combined with a lack of comprehensive reporting and appeals processes, has hindered evidence-based reforms.^9,10^

This study aimed to identify and categorise the different palliative and end-of-life care delivery mechanisms for people in prison across high-income countries, using a scoping methodology. We synthesised evidence from academic and grey literature, comprehensively delineating prison palliative care models in high-income countries to identify and share innovative practices and determine knowledge gaps.

The review builds on and extends previous research that defined prison hospice characteristics within the USA and examined the challenges of implementing community hospice standards within prisons, from a range of stakeholder perspectives,^9,10^ but did not delineate different models of care. This paper contributes to existing literature by offering a typology derived from international studies, broadening the predominantly USA-focussed evidence base.

Previous reviews were either limited to the USA and the UK,^11–14^ or excluded grey literature.^3,5^ Independent, third sector and charity organisations work extensively in the prison sector, so the inclusion of grey literature is essential to reflect current practice. The European Association of Palliative Care Task Force recently conducted a comprehensive mapping survey of palliative care within prison settings.^1^ This survey highlighted the importance of comparative analyses of care delivery approaches such as that reported here.

## Methods

We followed the five stages of Arksey and O’Malley’s scoping review methodology: specify the research question, identify relevant literature, select studies, map the data and synthesise the results.^15^ This approach is appropriate when evidence is emerging and disparate,^15^ and when attempting a thorough overview of the literature.^16^

Narrative synthesis was used to address step five in of Arksey and O’Malley’s methodology, the collation and summary of the data. Narrative synthesis was selected owing to the heterogeneity of included studies and grey literature. We followed Popay et al’s.^17^ approach, involving three iterative stages (developing a preliminary synthesis, exploring relationships within and between studies, assessing the robustness of the synthesis).

The review protocol was devised and reported according to the Preferred Reporting Items for Systematic reviews and Meta-Analyses extension for Scoping Reviews (PRISMA- SCR),^18^ prospectively registered in the Research Registry (Unique ID number: reviewregistry1260) and published open access.^19^

### Eligibility criteria

The Setting, Perspective, Intervention, Comparison, Evaluation (SPICE) framework was used to frame the research question due to its applicability in broader evaluations, that encompass setting and stakeholder perspectives.^17^

Inclusion and exclusion criteria are summarised in Table 1.

**Table 1.** Inclusion and exclusion criteria for study selection.

	Inclusion criteria	Exclusion criteria
Study participants	People in prison, families, carers, staff and volunteers who have experience of palliative care for people in prison	Families, carers, staff and volunteers who have experience of palliative care deliver not to adult people in prison
Communication or intervention	Studies reporting models and mechanisms of palliative healthcare in prisons, including perspectives of people in prison, families, carers, staff and volunteers; conducted in high-income countries	Studies reporting chronic or life-limiting illness without describing the care model, non-prison institutions or those not catering to adult people in prison and studies focussing on specific components of palliative care without describing the overall care delivery model
Type of study	Any study reporting original, empirical data, regardless of study design.	Case reports, protocols, editorials or commentaries.
Language of study report	English	Not reported in English
Timeframe	Studies published from 1 January 2000 until the search date (15 March 2023).	Studies published before 2000.

### Search strategy

In collaboration with a subject information specialist, the following databases were identified and searched: MEDLINE, EMBASE, CINAHL, PsycINFO and the Social Sciences Citation Index. The Medline search strategy (Table 2) was adapted for other databases (see Supplemental Material Files). All databases were searched on 15th March 2023 from 1st January 2000 to date, with results limited to the year 2000 onwards to ensure relevance to the current regulatory and policy context.

**Table 2.** Sources of grey literature.

Website/database
1. National Prison Hospice Association
2. International Association for Hospice and Palliative Care
3. Penal Reform International
4. Marie Curie
5. World Health Organisation (WHO)
6. Hospice UK
7. Google search (with country qualifier and/or advanced search function)
8. PROQUEST

The search results were collated and de-duplicated (EG) in Covidence.^20^

### Grey literature

The inclusion of grey literature is recognised as a key component of a comprehensive review process,^21–27^ but scoping reviews often lack detailed search strategies for grey literature, hindering replicability.^21^

In this study, grey literature was collected by searching relevant websites and databases, including ProQuest and Google. Table 2 includes all websites/databases searched. Subject-matter experts were consulted to help design the search strategy and identify any key material (see Acknowledgements).

Grey literature searches utilised combinations of the most common academic database keywords.

As of November 2020, 77 countries were classified as high-income by the World Bank.^19^ Following preliminary searches that suggested potential research activity, grey literature published in English was sought from the USA, Germany, UK, France, Canada, Australia, Netherlands, Sweden, Switzerland, Norway, Ireland, New Zealand and Iceland. A country qualifier was used in Google searches where feasible (e.g. ‘site:.au’ when searching for papers published in Australia). Not all countries have a qualifier; in this instance the advanced search in Google was used and the region-limiter function applied. Due to limitations in funding and time, the search was limited to the first four pages of Google results.

### Selection process

Titles and abstracts of identified records were screened by the primary researcher, with a second researcher independently screening a random 25% of the results. Any discrepancies were discussed within the research team. Study authors were contacted for additional information or to obtain full datasets where needed.

### Data extraction

Full-text articles were imported into Covidence for data extraction using a piloted template. The pre-piloted data extraction template (see Supplemental Material File) captured study design, country, information on care delivery (including providers, care settings, consideration of social/psychological/spiritual needs, admission criteria, training provided to staff and volunteers, multidisciplinary collaboration, older prisoner provision, interventions, prison policies, family rights, bereavement support and funding implications) and key outcomes.

A second reviewer reviewed extracted data from all papers with any discrepancies resolved by discussion.

### Quality appraisal

The Mixed Methods Appraisal Tool (MMAT) was used to assess included studies.^28^ The MMAT incorporates descriptive criteria for evaluating mixed-methods studies and has been widely tested for content validity. The 2018 MMAT version does not have a scoring function but proposes researchers present a robust rationale for the ratings of each criterion.

Records were assessed independently by two reviewers using the MMAT and a rating of ‘high’, ‘moderate’ or ‘low’ quality was agreed between them. For mixed-method studies, each component was assessed separately, and the lowest quality score was used. Publications assessed as high quality were considered more credible and relevant than those assessed as low or medium quality, but given the diffuse nature of evidence identified, low-quality evidence was also included.

### Narrative synthesis and typology development

The stages of narrative synthesis, as outlined by Popay, were adapted for this review.^17^ Through data extraction, appraisal and consensus with a second reviewer, studies with similar components, such as location of care, were grouped to develop a preliminary synthesis and derive the typology, with distinguishing properties as qualifiers for taxonomic differences.^29^ We analysed evidence iteratively to identify the typology, describe the models and highlight their outcomes, facilitators and challenges. Thematic analysis was used to formulate a preliminary synthesis, map key concepts and explore relationships between studies.^17^ A summary table with paper characteristics and summarised data is provided in Supplemental Materials. Grey literature provided useful context; in addition, findings relevant to the typology were integrated within the narrative.

## Results

**Figure 1.**
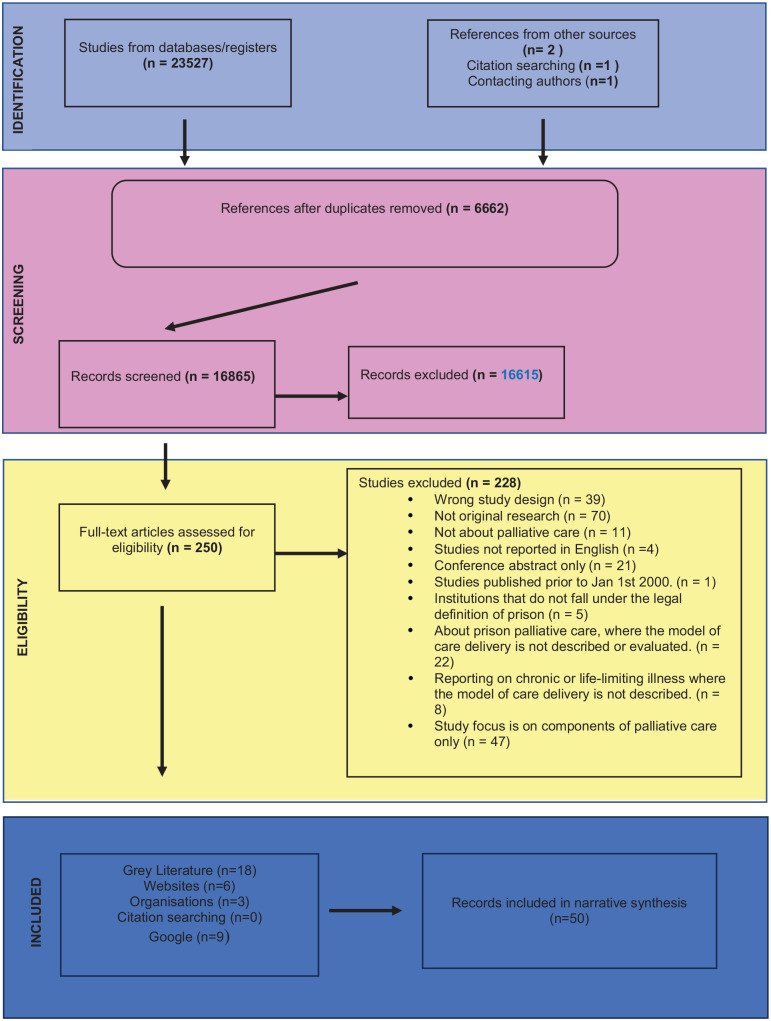
PRISMA flow diagram.

After deduplicating records, searching across databases, manual searches and contacting authors, a total of 16,865 records were identified; 16,615 were excluded at title and abstract screening (Figure 1). Two hundred and fifty full-text articles were screened; 22 reports in peer-reviewed journals and 18 grey literature publications met the eligibility criteria and were included in the synthesis. This included one additional paper^30^ and Supplemental Material Data from another study^1^ obtained from study authors.

### Characteristics of included evidence

Fifteen qualitative, six mixed-methods and one quantitative study were included in the synthesis. Studies were conducted between 2000 and 2022. Thirteen studies were from the USA,^11,12,30–40^ five from the UK,^8,41–43^ including Scotland,^44^ with individual studies from Canada,^45^ France^46^ and Australia,^47^ as well as a survey study from eight countries.^1^ Included studies and their key findings are presented in Table 3.

**Table 3.** Summary of included studies.

Study/Country	Design	Typology and theme	Outcomes	Facilitators	Challenges	Participant numbers and characteristics	Study quality using MMAT
Hoffman et al., 2011 (USA) *Characteristics of Prison Hospice Programs in the United States*	Quantitative cross-sectional	Embedded Hospice *Humanizing the Prisoner*	• Makes prisons humane. • Reduction in healthcare costs.	• Involvement of family • Paradigm shift to hospice culture through training • Inmate caregivers were key at 95% • Cessation of curative treatment is central.	• Management of pain (opiates) • Family depends of visitation procedures • Low % of community volunteers (41%) • Cessation of curative treatment -50% reported that this was essential for care	• 43 prison hospices were surveyed (survey completed by either prison warden, medical director or hospice coordinator).	• High Quality
Papadopoulos et al. 2016 (UK) *Current and Emerging Practice of End-Of-Life Care in British Prisons: Findings from an Online Survey of Prison Nurses.*	Mixed Methods	Community Collaboration *Building Relationships*	Ensuring prison nurses have relevant expertise may enable more people in prison to die in prison among familiar people, if this is their wish.	• Communication and links with community hospice • Availability of specialist equipment and changes to the environment (e.g. older persons wing) • Policy change for example, having an EOL policy and 24 h unlock • Inmate and prison staff support (buddies)	• Environmental barriers • Not having a palliative care/EOL care suite • Lack of a hospital wing • Lack of a quiet environment • The prison regime • Visiting restrictions • Lack of flexibility and privacy • Absence of special facilities for close relatives/friends to use on extended visits. • Lock down periods. • No open-door policy • Lack of willingness from senior staff to recognise that a terminally ill prisoner would no longer be a threat to society	• 31 respondents • (81% female) currently or previously employed in prisons across all regions of England, except the East of England.	• Medium Quality
Brooke Cooley Webb et al.2023 (USA) *Dying in Prison: End-of-Life Care Services in A State Correctional Facility*	Qualitative using semi-structured, one-one interviews	Embedded Hospice *Humanizing the Prisoner*	• Start to use inmate volunteers • Concerns about sexual assault ‘inmates are touching other inmates’ • Site will need to expand to keep up with demand	• Start to use inmate volunteers • Provide special training	• Lack of resources for medical staff or training provided to correctional officers • Lengthy wait for medical staff to receive medication/results • High staff turnover, some staff didn’t understand protocol which affected care	• 17 participants (correctional administrators, medical professionals and correctional officers) in a state 27-bed unit for prisoners at end-of-life.	• High Quality
Bronstein (USA) *The Impact of Prison Hospice* *Collaboration Among Social Workers and Other Professionals in a Criminal* *Justice Setting that Promotes Care for the Dying*	Qualitative design, telephone surveys and interviews.	Embedded Hospice *Humanizing the Prisoner*	• Transformational role • The respect and role people in prison have as part of the IDT, as well as in their hands-on work with the dying patients. • Productive collaborative and caring environment of hospice impacted prison community. • ‘More humane’	• High quality collaboration within the team with good diversity, person centred, social workers plays a coordinator role, support from warden • Prisoner volunteers’ impact on dying person - high quality care, dignity and respect, equity	• Sometimes hampered through poor coordination and lack of focus on outcomes for patients, lack of support from correctional staff • Needs more support from NPHA and community hospice programmes. • Earlier identification and referrals of people in prison requiring hospice required, especially from other prisons in the state that don’t have a hospice.	• 14 prison coordinators from 11 different states with diverse professional backgrounds (social work, nursing, psychology, business, law enforcement and chaplaincy).	• Medium Quality
Byock et al. 2006 (USA) *Promoting Excellence in End-of-Life Care: A Report on Innovative Models if Palliative Care.*	Mixed Methods	Embedded Hospice *Humanizing the Prisoner*	• Development and implementation of care standards was the primary mechanism for clinical care improvement. (The GRACE project) • Fostered cultural change in the institution marked by openness and interest in pain management • Implementing mechanisms to identify patients in need of comprehensive palliative care	• Education can increase quality and access to care, but only effective when clinicians have a desire to learn	• Palliative care programmes - increased chance of succeeding when housed in stable institutions • Data collection of outcome measures/patient and satisfaction were difficult	• 22 projects offering services across diverse settings, such as prison inmates, military veterans, renal dialysis patients and individuals with serious mental illness.	• High Quality
Chassagne et al. 2017 (France) *The Collision of Inmate and Patient: End-of-Life Issues in French Prisons.*	Qualitative	Outsourcing Care *Missed Opportunities*	• Concerns about inequality re: timely procedures, treatment and pain management • Focus on compassionate release by healthcare practitioners means that opportunities to provide PC are missed.	• Adjusting day-to-day practices for example, nurses ask the guards not to lock the doors.	**Challenges of UCSA model** (each prison has its own health care unit affiliated with a local hospital): • Lack of timely medical attention, no staff at weekends, nursing staff can’t provide social care support in prison, lack of equipment, staff suspicious of people in prison, patients suspicious of healthcare. • Limited relationship between inmates, caregivers and families **Challenges of UHSI model** (Prisoners requiring stays exceeding 48 h are transferred to either a local hospital or one of eight secure inpatient care units in university hospitals): • Caregiver–patient relationship suffers. • Not sufficient time and space necessary for care	• 14 inmates requiring PC, (average age of 59 years). • 70 semi-structured interviews with inmates, professionals and families.	• High Quality
Cichowlas et al. 2009 (USA) *Volunteer People in Prison Provide Hospice to Dying Inmates*	Descriptive Qualitative	Embedded Hospice *Humanizing the prisoner*	• ‘Life changing’ for prison volunteers • End-of-life inmates generally can get their needs fulfilled to the maximum degree.	• Strategies of flexible dates and times. • Flexibility - Family have adjusted visiting hours to suit • Adopts an adjusted definition of family • Both programmes run by full-time prison staff with additional duties assigned to the hospice programme.	• Difficulty of convening interdisciplinary team meetings.	• Five key components of prison hospice programmes were identified, assessing them within the specific context of the Dixon Correctional Center in Illinois.	• Low Quality
Cloyes et al.2016 (USA) *Essential Elements of an Effective Prison Hospice Program.*	Qualitative study utilising audio-recorded interviews, informal conversations, observations during site visits.	Embedded Hospice *Humanizing the Prisoner*	• Staff reported better at roles, changed prison culture	• Patient- and family-centred care • Formal MDT approach • Inmate volunteer programmes with training and support through ongoing meetings /supervision • A dedicated hospice coordinator • A volunteer coordinator (in the case of LSP, this is the same person as the hospice coordinator) • A primary nursing model. • The provision of 24-h presence and support at time of death through hospice vigil. • Education of correctional staff regarding the hospice mission and policies.	• Security first - limited quality of care staff could provide • Lack of hospice training for CO’s • A lack of shared values and culture	• 43 participants, including correctional officers, medical and nursing staff, hospice administrators and inmate hospice volunteers.	• High Quality
Cloyes et al. 2017 (USA) *Caring to Learn and Learning to Care: Inmate Hospice Volunteers and the Delivery of Prison End-of-Life Care.*	Qualitative – Focussed Ethnographic approach	Embedded Hospice *Humanizing the Prisoner*	• The attraction of replacing paid staff with cheaper—even unpaid–labor. • Use of peer-care volunteer programmes to meet these needs also raises questions as to whether this use of human capital is feasible or ethical.	• Combination of formal and informal training, education, mentorship and volunteer-staff interactions.	• Peer-care volunteer programmes raise questions as to whether the use of human capital is feasible or ethical. • Correctional administrators express concern regarding interactions among inmates that transgress institutional codes such as those that prohibit close personal contact between inmates. • Potential for victimisation inherent in placing vulnerable and sick inmates in the care and control. • Correctional nursing staff may be concerned about a ‘de-skilling’ of nursing care.	• 43 participants including correctional officers, medical and hospice staff and inmate hospice volunteers within LSP’s prison hospice programme.	• High Quality
Depner et al. 2018 (USA) *‘People don’t Understand What Goes on in Here’: A Consensual Qualitative Research Analysis of Inmate-Caregiver Perspectives on Prison-Based End-of-Life Care.*	Qualitative	Embedded Hospice *Humanizing the Prisoner*	• Dying inmates receive access to EOL care • Inmate-caregivers rehabilitate and given opportunity to provide nurture • Relieving care burden of correctional medical staff (reduce workload, stress and burnout) • Benefit to the community as a whole both inside and outside of prison - EOL care model may create a societal shift to help bridge the gap between EOL care for the privileged and EOL care for all • Facilities are faced with shrinking economic resources, increased age and illness of inmates and a lack of public support, making it difficult to meet EOL care mandates, this model helps the facility meet EOL mandates	• Using inmate-caregivers to improve support may also reduce workload, stress and burnout for medical staff, which are realities within the correctional system.	• Some inmate-caregivers acknowledged the controversial nature of a prison-based EOL programme, noting that not all correctional staff like/agree with the programme.	• 22 male inmate-caregivers from a single correctional facility EOL programme	• High Quality
Loeb et al. 2013 (USA) *Care and Companionship in an Isolating Environment: Inmates Attending to Dying Peers.*	Qualitative descriptive	Embedded Hospice *Humanizing the Prisoner*	• Transforming self through caring for others. • Inmates witness ‘humanity and *caring within the prison context, they likely will perceive the system as working for them’*.	• Staff support - practical and emotional • Support from fellow volunteers • Personal contexts influence people in prison in getting involved, living the role and transforming self through caring for others.	• Security issues • Lack of compassion • Poor quality of cleaning material • Lack of training • Lack of debriefing meetings • Lack of opportunities to memorialise the deceased, grief counselling • Lack of continuing education for the inmate caregivers & staff • Limited ability to provide a comforting environment	• 17 English-speaking male prisoners, aged 18 years or older, from four male population State Correctional Institutions (SCIs) of varying security levels in a mid-Atlantic state. Participants were either inmate workers or volunteers caring for peers with advanced stages of chronic illness.	• High Quality
McParland et al. 2021 (Scotland) *Caring, Sharing, Preparing and Declaring: How do Hospices Support Prisons to Provide Palliative and End of Life Care? A Qualitative Descriptive Study Using Telephone Interviews.*	Qualitative descriptive	Community Collaboration *Building Relationships*	• Typology is of international relevance • Further research is required to evaluate whether hospices meet the needs of those who die in Scotland’s prisons. • Recognition that as many as 82% (*n* = 14) of hospices are currently involved with prisons in some capacity. • Sharing knowledge and communication between prisons and hospices will be key to ensure expertise is shared on a national level. • Establishing robust networks of prisons and hospices will aid the development of services for this population.	• Supporting hospice staff who are caring for people in the CJS and preparing them properly (if care delivered in hospice) • Those actively involved in caring described a complex process of relationship building, staff preparation, effective communication and clear oversight • Geographical proximity • An understanding of specialist palliative care and the demands of custodial environments • Providing support over the telephone on an as-required basis may be sufficient for some	• Clearing security and moving about the prison • Physical environment strict regime • Administration of controlled medicines • People who want to die in prison are prevented from doing so as specialist equipment can’t be accommodated	• 17 hospices identified through the Scottish Partnership for Palliative Care. One representative (typically the CEO), provided information on their involvement with prisons.	• Medium Quality
Panozzo et al. 2020 (Australia) *Complexities and Constraints in End-of-Life Care for Hospitalized Prisoner Patients.*	Exploratory qualitative study	Outsourcing Care *Missed Opportunities*	• Recognition of need for improved clarity around application for compassionate release • Which key stakeholders should be involved, at what time in illness trajectory • Opportunity for the development and maintenance of in-reach IDT	• Not described	• Health care practitioners did not want knowledge of a patients’ crime, security level, or length of sentence to impact care • Issues of privacy • Continuity of care including pain management, from hospital to prison • Institutional barriers to effective pain and symptom management • Prison staff concerns about drug misuse/diversion • Constraints on timely breakthrough pain medications • Clinicians confused about guidance and practice	• 54 healthcare professionals actively caring for prisoner patients in a hospital setting, purposively sampled across various clinical disciplines and employment roles.	• High Quality
Prost et al 2020 (USA) *Characteristics of Hospice and Palliative Care Programs in US Prisons: An Update and 5-Year Reflection.*	Mixed Methods	Embedded Hospice *Humanizing the prisoner*	• Peer caregivers as the greatest strength of their programmes. • Peer caregivers reported ‘giving back’ -develop family-like connections and empathy for others. • Patients had usually been in the prison for extended periods of time and developed friendships with peer caregivers that make their death less challenging to come to terms with.	• IDTs remain integral to care • Peer caregivers continue to support dying patients	• Perceived public support for these programmes remains low • Reduced enthusiasm for the programmes may negatively influence administrative decision-making and programme resources.	• 113 EOL care prison facilities were surveyed, with 33 completing the survey. • Most facilities reported service for 5 or more patients daily, with 24 serving men, 5 serving women and 3 serving both men and women.	• Low Quality
Steely Smith et al. 2022 (USA) *‘We are all Humans and Deserve a Decent Way to Go’: Examining Professional’s Experiences with Providing End-of-Life Care in Correctional Institutions.*	Qualitative	Embedded Hospice *Humanizing the Prisoner*	• High job satisfaction was reported – ‘taking care of inmates is a niche’ • Some respondents saw their work as a ‘higher calling’	• Majority medical staff found correctional medicine more satisfying • Correctional staff prefer to work with older inmates (less risk) • CO’s belief that regardless of crime - they deserved to die with the same privileges as others	• Lack of resources, difficulties balancing care • Diff pain management for substance abusers • Security procedures • Lack of training and communication • Emotional stressors - maintaining professional relationships with inmates, dealing with manipulation and death and dying.	• 17 participants, including correctional and medical staff in an EOL care unit serving prisons in a southern state.	• High Quality
Turner et al. 2021 (Australia, Belgium, Czech Republic, England & Wales, France, Portugal, Scotland and Slovakia*)* *Mapping Palliative Care Provision in European Prisons: An EAPC Task Force Survey.*	Mixed Methods	Outsourcing Care *(Missed Opportunities, not including England & Wales who are developing their own PC provision in some cases)*	• Equity of access currently not happening in some countries. • May impeded early release, as prison authorities may be less willing to consider this.	• Not reported	• Little evidence of specific palliative care provision for people in prison established. • No prisons specifically for older people, no evidence of specialist provision for this age group	• Eight countries participated in the survey: Australia, Belgium, Czech Republic, England & Wales, France, Portugal, Scotland and Slovakia, with Steering Committee members in each country handling data collection.	• High Quality
Turner et al. 2011 (UK) *Care or Custody? An Evaluation of Palliative Care in Prisons in North West England.*	Mixed Methods	Community Collaboration *Building Relationships*	• One case study documents a prisoner who was released only to wish he had remained in familiar environment to receive treatment	• Strong links between prisons and hospices were developing	• Contrast between the philosophy and environment of hospices and that of prisons. • Lack of training for prison staff • Attitude of some palliative care staff raised concerns about putting time and resources into improving end-of-life care in prisons	• 27 staff members participated in the evaluation. 18 were healthcare staff from six prisons and 9 members of specialist palliative care staff from four local hospices.	• High Quality
Turner et al. 2017 (UK) *Palliative Care in UK Prisons: Practical and Emotional Challenges for Staff and Fellow People in Prison.*	Qualitative	Community Collaboration *Building Relationships*	• Continued increase in ageing population in prison - Continued challenge of balancing security with humanity	• HMPS has begun to respond to recommendations of the House of Commons Justice Committee (2013) • Some prisons have developed palliative care suites by converting cells to make room for hospital beds, hoists and other equipment and family room/bathroom	• Wide variations among prisons • Ministry of Justice has yet to produce policy guidance on palliative care provision across the whole service. • Need for both training and support if prison officers and health care workers are to be expected to cope with the emotional challenges.	• Prison staff (health care staff, prison officers and others) and prisoners.	• Medium Quality
Turner et al. 2018 (UK) *Ageing and Dying in the Contemporary Neoliberal Prison System: Exploring the ‘Double Burden’ for Older People in Prison.*	Mixed Methods – participatory action research	Community Collaboration *Building Relationships*	• To stakeholders - changes in policies and practice for older people in prison might impact on who is sent to prison • Sentencing practices, sentencing length, compassionate release and potential for providing specialist service for older and frail people in prison. • Creation of community based solutions for people in prison who require care more than punishment. • Large scale imprisonment means reduced access to healthy resources, food, healthcare, meaningful work or recreation, the result is a widening of the gap between the prison and wider populations. • Prison staff are bearing consequences of decisions around management of ageing and dying people in prison.	• Neoliberalism and the treatment of the sex offender diverts divert attention which might otherwise be paid to the structural roots of criminality.	• Benchmarking produced a staffing crisis. • Increase in historic offences. • Inadequacy of prison buildings designed for young, able men. • Reduction in officer numbers • Unevenness of healthcare provision • Escalating costs • Scant sympathy for sex offenders.	• 64 participants across various roles and settings inside the prison. Outside the prison, a palliative care consultant, 3 hospice nurses and 1 coroner. • 127 older prisoners, aged 55 years and above (a quarter were aged 70 years or older).	• High Quality
Wright et al. 200 (USA) *An Organisational Analysis of Prison Hospice.*	Qualitative	Embedded Hospice (Humanizing the Prisoner)	• Volunteers become best ambassadors for the programme • Volunteering ‘transformative’ influence on participants. • Rise in self-esteem and self-worth • Hospice resulted in a more caring and compassionate correctional environment	• Prison volunteers are vital • Careful planning and development critical to the integration of hospice into prison setting • Training, initial and ongoing, is essential. • Education of other personnel • Administrative support • Buy-in of correctional services personnel • Operates in an interdisciplinary fashion	• Correctional staff’s lack of support / knowledge of programme • Structure so all disciplines have input in the process of building and conducting a quality programme.	• 14 prison hospice programmes, with participants heading each unit; included 5 social workers, 3 chaplains, 2 health administrators, 2 nurses, 1 clinical psychologist and 1 nonprofessional correctional staff member.	• High Quality
Yampolskaya et al. 2003 (USA) *Hospice Care in Prison: General Principles and Outcomes.*	Qualitative	Embedded Hospice *Humanizing the Prisoner*	• Inmate volunteers transformed • Potential positive impact on overall inmate population. • The dying people in prison’ experience of comfort care was improved.	• Inmate volunteers • Cost-effectiveness • Adding security officials to the hospice teams	• Combining comfort care with correctional goals • Allowing relaxed rules for the dying patients while assuring security • Focussing on the patient and the family as the unit of care.	• 10 prison hospices, interviews with prison hospice providers in state and federal correctional institutions.	• High Quality
Shaw et al. 2021 (Canada) *‘Dying with a Smile, Just Knowing that Somebody’s Listened to Me’: End-of-Life Care and Medical Assistance in Dying in Canadian Prisons.*	Qualitative (document review and interviews)	Embedded Hospice Humanizing the Prisoner	• People in prison mistrust -believe that parole revoked due to lack of support if he was to be released with health conditions. • Dying often outsourced to hospital. • Limited resources on the outside lead to apprehension of release. • Suicide linked to slow process for compassionate release. • Trauma experienced by peer caregivers.	• Pal caregivers can walk around inmate population. • Pals developing familial bonds with patients. • Relationships were crucial factors in these men’s positive descriptions of healthcare. • Staff: ‘You’ve got some remarkable people here…Some of them are outstanding human beings and *professionals’.*	• Pal caregivers don’t get a break from work. • PI/RTC subordinates the clinical needs of those in its custody to security needs. • Lack of resources (poor quality environment etc.)	• 9 men serving long-term sentences at Pacific Institution/Regional Treatment Centre (PI/RTC) in Abbotsford, BC.	• Medium Quality

EOL: end of life; MDT: multi-disciplinary team; PC: palliative care; HMPS: His Majesty’s Prison Service

### Quality of studies

The methodological quality of the studies was mixed (see Supplemental Materials of the 22 studies, 15 met all appraisal criteria). Due to inadequate reporting, five were considered at medium risk of bias and two at high risk.

### Grey literature

#### Typology of models of palliative care delivery

This scoping review identified three models of palliative care for people in prison in high-income countries: ‘Embedded Hospice’, ‘Outsourcing Care’ and the ‘Community Collaboration’ model.

##### Embedded hospice model

This model is typified by an interdisciplinary team (IDT) and volunteer caregivers providing care on-site.^11,12,30–32,35–39^ Thirteen out of 14 included studies reporting this model were from the USA. Studies were mostly high quality, although two were deemed medium quality and two were low. A survey identified 113 functioning prison hospices in the USA,^12^ but the implementation of these programmes varied.^12,33^ Two papers (from one study) describe a regional healthcare resource, serving all people in prison within that American state.^30,31^ One Canadian study showcased the ‘Pal Program’, providing companionship for people in prison with serious illnesses.^45^

All 13 USA studies reported inclusive IDTs with medical professionals and prison staff.^12^ Data indicates that as early as 2011, 94% of surveyed hospices in the USA utilised unpaid peer caregivers.^11^ Chaplains, social workers and inmate caregivers in the IDT primarily aided families by providing psychoeducation about dying and promoting familial reconciliation.^33,38,35^

Admission criteria for utilising the service varied between the 13 studies from the USA; from a prognosis of either 6^30^ to 12 months^11,40^ to no requirement of time-to-death prognosis.^29^ Three studies reported a requirement for a do-not-resuscitate (DNR) order, while others didn’t; the reason for this variation is unclear.^26,34,35^

Where compassionate release mechanisms were reported, pre-release planning involved the reinstatement of an individual’s benefits and organisation of their community care,^10^ with most applications led by social workers.^32^ Medical Assistance in Dying (MAiD) has been legally available in Canada since 2016.^45^ Incarcerated patients encounter difficulties in equitable access to MAiD services. The cited Canadian study reveals challenges in accessing early release for people in prison, highlighting concerns that those with terminal illness may choose MAiD over preferred community-based palliative care due to such obstacles. These difficulties can contribute to heightened suicidal ideation, amplified by uncertainties about supportive medical staff and fears of repercussions for inquiring about MAiD.^45^

The grey literature illustrated the rise of the Embedded Hospice model in the USA. The Humane Prison Hospice Project report cited low recidivism rates for inmate volunteers^48^ and evidence of the transformative potential of inmate caregiving. The report includes accounts of hardened gang members demonstrating empathy towards the terminally ill,^49^ and murderers with 50-year sentences finding redemption through caring.^50,51^

### Psychosocial support

Psychosocial support varied, with psychiatrists and psychologists included in only some IDTs.^33^ One study found psychologists in only 7% of programmes surveyed in the USA.^34^ Counselling by chaplains, social workers and peer caregivers was common, with volunteers also trained in psychosocial aspects of dying.^35,36^ Peer caregivers formed close bonds with patients, offering companionship and comfort.^33,34^ Chaplains were the main providers of spiritual care,^29,35–37^ although peer caregivers, might read religious texts ^11,38,45,39^ and provide comfort through prayer.^34^

### Social care

Informal companionship (facilitated by peer caregivers) often went beyond assisting with daily activities to attending to an individual’s unique wants and needs.^26,29,36,45^ These relationships might offer protection, safeguarding people in prison from potential abuse.^37^ Four studies did not report social care provision.^33–35^

### Rights of families

Visitation policies generally permitted non-incarcerated families and friends to visit, with some programmes facilitating videotaped messages.^12^ Flexible visiting arrangements were emphasised,^33,35^ and the definition of family extended to include fellow inmates, reducing barriers associated with strict protocol adherence.^35^

### Bereavement support

Bereavement support involved regular meetings or ad hoc support.^29^ Informal discussions with staff, memorial services and peer mentorship were observed.^33,35–37^ Bereavement support could take the form of signposting to bereavement services, condolence cards, telephone calls or psychoeducation on the stages of dying and anticipatory grief.^35^ Some programmes surveyed lacked opportunities to memorialise the deceased or access grief counselling.^12^ The Canadian study highlighted challenging conditions faced by inmate volunteers, leading to emotional distress and potential Post Traumatic Stress Disorder following a death of a fellow inmate.^45^

### Funding implications

This model reports cost-effectiveness due to reduced hospital transport and DNR orders, reducing the cost of life-sustaining interventions.^29,36^ In the Canadian study, prison caregivers acknowledged low compensation in their work ($25 every 2 weeks on the PAL programme).^45^ In contrast, a study in the USA found stable expenses despite high healthcare costs in the last 6–12 months.^33^

### Facilitators and challenges

This model appears to be supported by several key facilitators, principally the inmate caregivers’ person-centred practice.^12,32,39^ The importance of education and training, whether through formal programmes or informal mentoring, for both staff and volunteers, was underscored.^33,34^ The collaboration of an IDT was found to be crucial,^11^ and the addition of a volunteer coordinator was positively noted.^35^ Programmes administered by full-time prison staff assigned additional hospice duties were deemed effective.^34^ Another important facilitator was the ability of families to feel included;^8,35–38,46^ however some people in prison regarded fellow inmates as their family.^37^

Prominent challenges in implementing this model included security considerations impacting opiate administration within prisons.^11,12,45^ Healthcare concerns were often overshadowed by security protocols, hindered by a lack of knowledge and training.^29,35^ Cultural differences between hospice and prison raised concerns about mistrust and potential exploitation.^35–37^ Inadequate resources for a comfortable environment and limited public support were common.^12,30,31,38^ Prison staff were reported to question requests for specific food items^12^ and be hesitant about relaxing visiting rules.^29,35^

The stability of the institution emerged as a potential challenge, with palliative care programmes identified as vulnerable during periods of infrastructural unrest.^33^ Additionally, the insistence on having DNR orders in place could undermine trust,^29^ or access to MAiD for Canada’s people in prison.^45^

The Embedded Hospice model is associated with a pervasive theme of ‘humanizing the prisoner’. This applies to the peer caregiver volunteers as well as the dying individuals, fostering transformative experiences for all involved. The evidence from the Humane Prison Hospice Project report underscores the profound impact of inmate caregiving, showcasing instances of empathy from hardened gang members and stories of redemption among people in prison serving lengthy sentences. The person-centred care reported in this model facilitates a humanising experience for all involved.

#### The ‘outsourcing care’ approach

The three studies reported under this heading, all deemed high quality, represent an emerging approach, in which end-of-life care for people in prison is largely provided outside of the institution. Exceptions are found in the European Association for Palliative Care (EAPC) survey,^1^ which reports some instances of palliative care suites in English and Welsh prisons, although they are in the minority.

The EAPC survey included Australia, Belgium, Czech Republic, England and Wales, France, Portugal, Scotland and Slovakia; other studies in this category were conducted in France^46^ and Australia^1,47^

Most countries offered primary healthcare within their prisons; however, in Portugal, 14 prisons out of 55 reported no prison healthcare units.^1^ In Australia, Belgium, the Czech Republic, France, Portugal and Slovakia, people in prison are typically transferred to different prisons or hospitals to receive care.^1,46,47^ Inmate volunteers are not commonplace, and IDTs consist of physicians, nurses and social workers.^1,46,47^

In France, prisoner preference for receiving care within a hospital may be one factor driving this model.^46^ Conversely, Australia faces challenges in providing diverse services across vast geographical areas, meaning people in prison may be transferred to a centrally located maximum security prison for hospital visits, regardless of their security status.^47^ However, evidence suggests that in England and Scotland, prisons are beginning to collaborate with local services to support patients who are not transferred. Currently, England and Wales have some dedicated palliative care units in a few prisons.^1^

Studies show how compassionate release policies affect service delivery. Chassagne’s 2017 study suggested that healthcare professionals favoured compassionate release as a suitable approach to palliative care, with dying individuals granted full ‘patient’ status.^46^ In Australia, jurisdictions decide to release cases individually,^47^ while Belgium involves a criminal court judge and written prisoner requests.^1^ England, Scotland and Wales share early release criteria for low re-offence risk, whereas Portugal, Slovakia and the Czech Republic lack specific early release policies.^1^

### Psychosocial support

Psychosocial support was not referenced in studies describing this model, despite people in prison feeling isolated and unsupported.^46^ The absence of comfort care like special food, therapies, music and cultural or religious practices, was noted due to prison protocols.^1^

### Social care

Some aspects of social care, such as meals or personal care, could not be provided outside the prison but also could not always be provided by the prison nursing staff.^1,46,47^ Consequently, the responsibility fell to fellow inmates, but care could be obstructed due to a lack of equipment (e.g. wheelchairs or specialist bedding) and an absence of specific policies relevant to people in prison’s palliative care needs.^1^ A lack of specialist equipment, mistrust between people in prison and healthcare staff, insufficient communication and inadequate social care compromised the quality of care.^46,47^ The European Association for Palliative Care survey report highlighted ‘life support workers’ employed in French prisons to assist older people in prison, and some separate units for older people in prison in England and Wales.^1^

### Rights of families

Limited family involvement, infrequent communication from physicians and restricted visits from families could hinder adequate preparation for death.^46^ Furthermore, secure ward settings were reportedly considered unsuitable for children to visit.^46,47^

### Bereavement support

Bereavement support was not reported in these studies.

### Funding implications

The funding implications of this model were largely not discussed. The European mapping study report noted only that funding for prison palliative care falls under each state’s responsibility.^1^

### Facilitators and challenges

Facilitators of delivering good end of life care via this model included prisons addressing the needs of their older and end-of-life population,^1^ and staff managing to influence routine practice.^46^ Prisons showed some flexibility regarding release policies to meet individual needs: people in prison in France have the option of requesting parole or electronic surveillance during treatment,^46^ and in England and Wales governors can grant temporary release on compassionate grounds, to be recalled if circumstances change.^1^

Several challenges were identified. Healthcare professionals can lack clarity on how to advocate for people in prison and support decision-making.^1^ A lack of guidance for healthcare staff regarding pain medication in the secure setting, limited access to patients’ rooms or delays in authorisations led to poor continuity and quality of care.^46,47^ Prison healthcare staff showed reluctance in seeking help or actively collaborating with the hospital palliative care team.^46,47^ This lack of communication and collaboration may be attributable to organisational constraints,^46,47^ resulting in limitations in comfort care provisions and family visitations.^1,46^ In cases of transfers to different security-level prisons, some people in prison chose to forgo healthcare to stay in their preferred residence, resulting in treatment delays.^47^ Due to the fragmented nature of care reported within this model, there were concerns about timely diagnosis.^1^A lack of initiatives was notable in Australia, Belgium, the Czech Republic, Portugal and Slovakia,^1^ with no evidence of specialist palliative care for older people in prison.

The overarching theme of ‘missed opportunities’ was identified in this emerging model. Psychosocial support is often unaddressed despite people in prison feeling isolated, and key aspects of comfort care such as, special food, therapies and attention to cultural practices, are unreported. Additionally, challenges to social care provision, compounded by environmental challenges and a lack of policies to ensure the palliative care needs of people in prison are met highlight the need for research documenting best practice. The absence of reported bereavement support further underscores the model’s potential to omit adequately addressing holistic end-of-life needs.

#### The collaborative community model

Five studies, all from Scotland and England, reported the collaborative community model, which involves prisons’ engagement with other healthcare facilities or practitioners to assist patients. Specifically, prison teams actively pursued multidisciplinary collaboration with local palliative care specialists.^44^ Staff from community palliative services, alongside prison healthcare personnel, offered care to people in prison through in-reach services.^8,42^ Some prisons had their own end-of-life care programmes, with a growing number of inmates trained as ‘buddies’ to support fellow people in prison.^8^

Central to this model is the idea that individuals in prison with serious illnesses should ideally receive care within or close to their ‘home’ institution, ensuring comfort and familiarity for the person in prison coping with serious illness. Three of the studies reporting this model were assessed as medium quality^8,44,41^ and two as high.^43,52^

Research documenting this model showed compassionate release was perceived as a complex procedure by community hospices.^44^ This might be attributed to stringent criteria, such as a requirement of less than 3 months to live, being met.^41^ It is suggested that shifting policies in the UK to prioritise palliative care facilities within prisons, rather than reforming compassionate release, presents new challenges for prison staff,^43^ a consideration for those adopting this model.

In the grey literature, a 2019 report by the Australian Department of Health highlighted barriers to palliative care in prison, recommending more formalised collaboration between prisons and community care providers.^53^ At the 2022 Conference on Correctional Health Care in Australia, the Network Palliative Care Model was introduced to provide tailored care to incarcerated patients and enhance clinician skills.^54^ In Scotland, a community collaboration model was reported between a local hospice and HMP Edinburgh.^55^ A UK study outlined three approaches to palliative care delivery: individual prison, cluster and regional, emphasising the need for an integrated model of care in UK prisons.^56^ Evidence from England illustrated notable examples of the model, including a Norwich prison with a 15-bed unit for older adults which provides care within the prison environment with support from hospice nurses and medical staff.^57^ Dartmoor Prison collaborates with the local hospice’s outpatient specialist palliative care nurses, providing personalised end-of-life care through nurse-led clinics and advice on in-cell adjustments.^58^

### Psychosocial support

There was considerable variation in prison staff’s self-reported ability to meet people in prison’ psychosocial and spiritual support needs.^42^ High levels of anxiety and vulnerability were reported among older people in prison, intimidated by younger inmates due to physical and mental frailty.^42^ People in prison feared receiving inadequate care, due to the perceived rigidity and unkindness of the system.^43^ One study showed that some Scottish hospices provided support to their local prison’s psychiatrist.^44^ There was some evidence that prison ‘buddies’ provided pastoral support, and in certain prisons, individuals were granted increased visitation privileges.^8^

### Social care

Even though some prisons have converted cells to accommodate beds, hoists and specialised equipment,^41^ the use of ‘buddies’ to deliver personal care amongst an older population of predominantly sex offenders is considered unsafe and inappropriate.^41^

### Rights of families

Certain inmates cultivate their closest bonds with fellow people in prison. ^41,42^ However, less than one-fifth of British prisons surveyed permitted visits from children towards the end of life.^41^ A noteworthy facilitator for this model was when hospices actively sought out family members to facilitate visits.^44^

### Bereavement support

Weaknesses in bereavement care were highlighted, linked to insufficient staff training.^42^ Family liaison officers described the ‘grim’ task of attending funerals, with little support for staff in this aspect of their work.^41^ A discernible contrast was noted in the training levels between hospice and prison staff.^41^ Chaplaincy was reported to provide care for both the dying person and their relatives,^42^ and to grieving people in prison after the death of an inmate through communal prayer.^41^ Spiritual care was offered via chaplains and prison psychiatry.^41,42,44^ Additionally, peer groups and staff offered emotional support outside of formal structures.^41,42^

### Funding implications

The funding implications of this model were not discussed in these studies.

### Facilitators and challenges

Some prisons had begun to develop palliative care suites, enabling some care to be delivered on-site.^41^ A UK study reported that environmental adaptations, like creating an older prisoner's wing and implementing the use of ‘buddies’, had been well received.^8^ Collaboration with nearby community palliative care services facilitated training and knowledge exchange.^44^ Hospices actively seeking family members to facilitate visits also utilised this model.^44^ Successful hospice care for prisoner patients or those released from prison requires staff to be ready, trained and aware of potential risks associated with the individuals’ criminal backgrounds.^44^

Barriers to the success of this model can be both systemic and environmental.

Typical prison cells are reported to be too small for hospital beds, and limited access to clean bedding, showers and clothing poses challenges in addressing social care needs for some people in prison.^41^ Establishing collaborative relationships between prisons and community palliative care providers relies on *‘a complex process of relationship building, staff preparation, effective communication, and clear oversight’*, where the balance between care and custody requires understanding both contexts and a willingness to adapt policies accordingly.^44^ Prison staff shortages significantly impact the time nurses can allocate to people in prison, especially those who are older with multimorbidity.^41,42^ Limited access to specialised equipment, restricted availability of pain relief and the absence of a peaceful environment exacerbate the challenging conditions for delivering palliative care.^41–43^ Moreover, in the UK only 53% of prisons have any palliative care policies, 17% lack policies and 30% are unaware of their policy status.^8^

The grey literature search yielded an additional 18 publications for inclusion, from eight countries (USA, Switzerland, Scotland, Ireland, Canada, Australia, New Zealand and the UK). There were eight web articles,^48–51,59–62^ two poster presentations,^55,56^ one conference abstract,^54^ two official reports,^63,64^ four policy documents^58,65,66^ and one charity publication.^57^

Findings from the grey literature highlighted two key themes illustrating policy and practice debates. The first concerned compassionate release, that is the process by which inmates in criminal justice systems may be eligible for immediate early release on grounds of particularly extraordinary or compelling circumstances, including facing imminent death,^9^ enabling access to palliative care services. There were reports of an increase in demand for compassionate release since the COVID-19 pandemic.^60^ Challenges were documented because policies often rely on a prognosis of 12 months or less, which can rarely be declared with certainty.^59^ For areas with a large older prison population like Massachusetts, where over 15% of inmates are over 55, this is a concern.^60^ A 2018 Irish investigation report following the death of a terminally ill prisoner recommended Compassionate Temporary Release as the preferred policy for terminally ill people in prison in Ireland, to avoid undue suffering.^63^

The second theme related to challenges in palliative care delivery. A Swiss report detailed insufficient staff training, an absence of relevant legislation, and conflicting priorities between punishment and rehabilitation.^61^ These were echoed in Canada, where the Office of the Correctional Investigator found deficiencies in provision such as limited family support, insufficient training and obstacles to round-the-clock care.^65^ In New Zealand, the case of Vicki Letele was widely reported: a 35-year-old mother diagnosed with terminal cancer during her sentence, and granted compassionate release following an appeal. The establishment of high-dependency units in prisons and compassionate release policies was anticipated to increase following the visibility of this story.^62^

Hospice UK’s 2021 report highlighted inequalities for people in prison in the UK accessing palliative care, proposing recommendations like standardised bereavement support.^66^ The Royal College of Nursing also called for improved end-of-life care across the prison estate.^64^ A central theme within the collaborative community model was ‘building relationships’ through prisons’ proactive engagement with community healthcare. These collaborations involve multidisciplinary efforts with local palliative care specialists, and some prisons have developed their end-of-life care programmes, training inmates as ‘buddies’ to support peers.

## Discussion

Embedded hospice models in the USA are prevalent and demonstrate promising evidence for enhancing the care experience for recipients and peer caregivers. Chaplains, social workers and peer caregivers provide psychosocial support, yet documented assessment and strategies for managing the distinctive needs of this group and their families are lacking, despite their acknowledged complexity.

### Main findings

In this comprehensive scoping review, we identified a typology of three models of care delivery for people in prison in high-income countries: (1) Embedded hospice model, typified by an interdisciplinary team and volunteer caregivers providing care on-site; (2) Outsourcing Care model, in which end-of-life care is provided outside the prison; (3) Collaborative Community model, which involves prisons engagement with other healthcare facilities or practitioners. End-of-life care provision in prisons in high-income countries also varies in the application of policies regarding Early Release on Compassionate Grounds, the consideration of which should form part of palliative care for a dying prisoner.

The USA primarily utilises the Embedded Hospice model with inmate caregivers, and there is some evidence that this model results in high-quality care, cost reduction and potentially transformative experiences for peer caregivers. Although the Canadian study also reports this model, the extent to which this model has been adopted across the country is unclear.

The voluntary status of peer caregivers should be highlighted, as their choice to take on this role underscores the potentially transformative benefits and relies on the quality of the relationships between caregiver and patient. Considering potential exploitation concerns, research is needed to understand the value of fostering these meaningful connections and the contextual factors that allow them to flourish.

The fragmented Outsourcing Care model risks missing timely diagnosis, effective pain management and family communication for those in prison. Evidence regarding psychosocial needs and bereavement support were generally lacking and hospital clinicians’ lack of information regarding prison protocols had a negative impact on holistic care.

Good practice in the Community Collaboration model depends on motivated staff and grassroots initiatives, where prison or community palliative care providers actively seek working relationships. However, not all prisons have palliative care policies, which could compromise staff’s incentive to implement pre-emptive planning by establishing relationships with relevant community services.

There is limited evidence regarding the psychosocial needs of people in prison facing serious illness and their management, including who is best equipped to provide psychosocial services to the dying in a prison context.^3^ Bereavement support was well documented in Embedded Hospice model studies, however, studies in the Collaborative Community model indicate insufficiencies in the support available. There is preliminary evidence of financial benefit within the Embedded Hospice model, but the cost-effectiveness of the other models has not been examined.

### What this study adds?

Previous systematic reviews synthesised evidence regarding different aspects of death, dying and palliative care within prisons,^3–7,13,14^ but either focussed solely on psychosocial care,^4^ or did not identify and describe different models.^5,7,11–14^ This review identifies and describes three broad typologies of palliative care delivery in prisons within high-income countries and their associated facilitators and challenges.

It reaffirms previous findings on inadequate specialised equipment, conflicting care and custody principles and the transformative impact of hospice care on volunteers.^2,5,6^ It further highlights the role of peer caregivers in delivering social care and reports available data on funding implications. Incorporating grey literature provides essential non-academic content that enriches our understanding of current policy and practice priorities.

Future research should prioritise studying countries where evidence is lacking, to enable comparisons and ensure equal access to appropriate care for incarcerated individuals, as well as systematically describe and assess palliative care models in prisons to establish a robust evidence base for service design and policy making. Research should assess the financial impact of inmate caregiver programmes, compare community versus prison hospice and different compassionate release policies, identify the psychosocial and bereavement support needs of seriously ill people in prison and their close persons within and outside the prison, and explore the ethical implications of inmates as volunteers, and the use of MAiD for people in prison.

In countries not implementing the Embedded Hospice Model, collaboration between hospitals, community palliative care and prison healthcare services appears crucial for managing people in prison’s end-of-life care. Developing local policies and sharing good practice could promote sustainability while ensuring compliance with frameworks (e.g. UK’s Dying Well in Custody Charter) would guarantee consistent adherence to quality standards.^67^ Strategic priorities include consulting on sentencing practices for older people in prison, optimising compassionate release policies and providing suitable provisions and trained staff for the ageing prison population.

### Strengths and limitations

Study selection used clear criteria and a systematic data extraction process for replicability. Quality appraisal was systematic and robust. Only English-language studies published since 2000, including grey literature, were included for contemporary evidence.

The use of Google poses limitations due to variable search results influenced by factors like location and search history. Despite attempts to mitigate bias through measures including clearing history and using incognito mode, complete elimination of bias remains a challenge. Due to time and resource constraints, only one screener was utilised in searching the grey literature, focussing on the initial four pages of Google results. The narrative synthesis was limited by study scope and heterogeneity, addressing various palliative care models. Families’ views were underrepresented in the majority of included studies. The articles that conducted interviews with incarcerated individuals were undertaken in male prisons only and not female prisons.

## Conclusion

In high-income countries, end-of-life care approaches in prisons vary significantly. The USA predominantly uses the Embedded Hospice model with inmate caregivers, which shows potential for quality care and cost reduction. Outsourcing end-of-life care can lead to fragmentation and missed opportunities for comprehensive care. The Community Collaboration model shows promise but relies on motivated staff and grassroots initiatives. Limited evidence exists regarding the psychosocial needs of seriously ill people in prison and the most suitable caregivers for addressing their potential complexities, and bereavement support is limited outside the Embedded Hospice model. Addressing these disparities is crucial for providing dignified and compassionate care to all incarcerated individuals nearing the end of life.

## Supplemental Material

sj-pdf-1-pmj-10.1177_02692163241242647 – Supplemental material for How do people in prison access palliative care? A scoping review of models of palliative care delivery for people in prison in high-income countries Supplemental material, sj-pdf-1-pmj-10.1177_02692163241242647 for How do people in prison access palliative care? A scoping review of models of palliative care delivery for people in prison in high-income countries by Emma Gilbert, Nick De Viggiani, Joana de Sousa Martins, Tanuka Palit, Jessica Sears, Daniel Knights, Audrey Roulston, Mary Turner and Lucy E Selman in Palliative Medicine

## References

[1] Turner M Chassagne A Capelas ML , et al. Mapping palliative care provision in European prisons: an EAPC Task Force Survey. BMJ Support Palliat Care. Epub ahead of print 22 April 2021. DOI: 10.1136/BMJSPCARE-2020-002701. 33888490

[2] Maschi T Richter M. Human rights and dignity behind bars. J Correct Health Care 2017; 23(1): 76–82. 28100140 10.1177/1078345816685116

[3] Johns L Weightman S Blackburn P , et al. A systematic literature review exploring the psychosocial aspects of palliative care provision for incarcerated persons: a human rights perspective. Int J Prison Health. Epub ahead of print 15 December 2021. DOI: 10.1108/IJPH-02-2021-0017 34902234

[bibr4-02692163241242647] Schaefer I DiGiacomo M Heneka N , et al. Palliative care needs and experiences of people in prison: a systematic review and meta-synthesis. Palliat Med 2021; 36(3): 443–461. 34965778 10.1177/02692163211068278

[bibr5-02692163241242647] McParland C Johnston BM. Palliative and end of life care in prisons: a mixed-methods rapid review of the literature from 2014–2018. Vol 9, p. e033905. British Medical Journal Publishing Group, 2019. DOI: 10.1136/bmjopen-2019-033905 PMC7008433 31874895

[bibr6-02692163241242647] Stone K Papadopoulos I Kelly D. Establishing hospice care for prison populations: an integrative review assessing the UK and USA perspective. Palliat Med 2012; 26(8): 969–978. 21993807 10.1177/0269216311424219

[7] Petreca VG. Death and dying in prison: an integrative review of the literature. J Forensic Nurs 2021; 17(2): 115–125. 33843810 10.1097/JFN.0000000000000318

[8] Papadopoulos I Lay M. Current and emerging practice of end-of-life care in British prisons: findings from an online survey of prison nurses. BMJ Support Palliat Care 2016; 6(1): 101–104. 10.1136/bmjspcare-2015-000880 26534855

[9] Handtke V Wangmo T Elger B , et al. New guidance for an old problem: early release for seriously ill and elderly prisoners in Europe. Prison J TA TT 2017; 97(2): 224–246.

[10] Handtke V Wangmo T. Ageing prisoners’ views on death and dying: contemplating end-of-life in prison. J Bioeth Inq 2014; 11(3): 373–386. 24965438 10.1007/s11673-014-9548-x

[11] Hoffman HC Dickinson GE. Characteristics of prison hospice programs in the United States. Am J Hosp Palliat Med 2011; 28(4): 245–252. 10.1177/1049909110381884 20834030

[bibr12-02692163241242647] Prost SG Holland MM Hoffmann HC , et al. Characteristics of hospice and palliative care programs in US prisons: an update and 5-year reflection. Am J Hosp Palliat Med 2019; 37(7): 514–520. 10.1177/1049909119893090 31808349

[bibr13-02692163241242647] Wion RK Loeb SJ. End-of-life care behind bars: a systematic review. Am J Nurs 2016; 116(3): 24–37. 10.1097/01.NAJ.0000481277.99686.82 26871892

[14] Maschi T Marmo S Han J. Palliative and end-of-life care in prisons: a content analysis of the literature. Int J Prison Health 2014; 10(3): 172–197. 25764177 10.1108/IJPH-05-2013-0024

[15] Arksey H O’Malley L. Scoping studies: towards a methodological framework. Int J Soc Res Methodol 2005; 8(1): 19–32.

[16] Peters MDJ Marnie C Colquhoun H , et al. Scoping reviews: reinforcing and advancing the methodology and application. Syst Rev 2021; 10(1): 1–6. 34625095 10.1186/s13643-021-01821-3 PMC8499488

[17] Popay J Roberts H Sowden A , et al. Guidance on the conduct of narrative synthesis in systematic reviews. In: A product from the ESRC methods programme Version. Vol. 1, 2006, p. b92. https://www.researchgate.net/publication/233866356

[18] Tricco AC Lillie E Zarin W , et al. PRISMA extension for scoping reviews (PRISMA-ScR): Checklist and explanation. Ann Intern Med 2018; 169(7): 467–473. 30178033 10.7326/M18-0850

[19] Gilbert E Turner M De Viggiani N , et al. Developing a typology of models of palliative care delivery in prisons in high-income countries: protocol for a scoping review with narrative synthesis. BMJ Open 2022; 12(4): e060886. 10.1136/bmjopen-2022-060886 PMC9058786 35487724

[20] Covidence systematic review software. Veritas health innovation, Melbourne, Australia. https://www.covidence.org (2023).

[21] Heath A Levay P Tuvey D. Literature searching methods or guidance and their application to public health topics: a narrative review. Health Info Libr J 2022; 39(1): 6–21. 34850535 10.1111/hir.12414 PMC9300102

[bibr22-02692163241242647] Briscoe S Abbott R Lawal H , et al. Feasibility and desirability of screening search results from Google Search exhaustively for systematic reviews: a cross-case analysis. Res Synth Methods.Epub ahead of print 19 January 2023. DOI: 10.1002/JRSM.1622. 36633509

[bibr23-02692163241242647] Mahood Q Van Eerd D Irvin E. Searching for grey literature for systematic reviews: challenges and benefits. Res Synth Methods 2014; 5(3): 221–234. 26052848 10.1002/jrsm.1106

[bibr24-02692163241242647] Paez A. Gray literature: An important resource in systematic reviews. J Evid Based Med 2017; 10(3): 233–240. 28857505 10.1111/jebm.12266

[bibr25-02692163241242647] Benzies KM Premji S Hayden KA , et al. State-of-the-evidence reviews: advantages and challenges of including grey literature. Worldviews Evid Based Nurs 2006; 3(2): 55–61. 17040510 10.1111/j.1741-6787.2006.00051.x

[bibr26-02692163241242647] Hopewell S McDonald S Clarke MJ , et al. Grey literature in meta-analyses of randomized trials of health care interventions. Cochrane Database Syst Rev 2007; 2: 3. 10.1002/14651858.MR000010.pub3 PMC8973936 17443631

[27] Mahood Q Van Eerd D Irvin E. Searching for grey literature for systematic reviews: challenges and benefits. Res Synth Methods 2014; 5(3): 221–234 26052848 10.1002/jrsm.1106

[28] Hong QN Fàbregues S Bartlett G , et al. The mixed methods appraisal tool (MMAT) version 2018 for information professionals and researchers. Educ Inf 2018; 34(4): 285–291.

[29] Stapley E O’Keeffe S Midgley N. Developing typologies in qualitative research: the use of ideal-type analysis. Int J Qual Methods 2022; 21: 5.

[30] Smith MK ten Bensel T. Dying in prison: end-of-life care services in a state correctional facility. Policing County Lines. Epub ahead of print 19 January 2023. DOI: 10.1007/978-3-030-54193-4.

[bibr31-02692163241242647] Steely Smith M Cooley B Bensel TT . We are all humans and deserve a decent way to go: examining professional’s experiences with providing end-of-life care in correctional institutions. Crim Justice Rev 2022; 47(2): 225–242.

[bibr32-02692163241242647] Bronstein LR Wright K. The impact of prison hospice: Collaboration among social workers and other professionals in a criminal justice setting that promotes care for the dying. J Soc Work End Life Palliat Care 2006; 2(4): 85–102. 17387096 10.1300/j457v02n04_05

[bibr33-02692163241242647] Byock I Twohig JS Merriman M , et al. Promoting excellence in end-of-life care: a report on innovative models of palliative care. J Palliat Med 2006; 9(1): 137–151. 16430353 10.1089/jpm.2006.9.137

[bibr34-02692163241242647] Cichowlas JA Chen Y. Volunteer prisoners provide hospice to dying inmates. Annals Health L 2009; 19: 127. 21495560

[bibr35-02692163241242647] Cloyes KG Rosenkranz SJ Berry PH , et al. Essential elements of an effective prison hospice program. Am J Hosp Palliat Care 2016; 33(4): 390–402. 25735806 10.1177/1049909115574491 PMC4558405

[bibr36-02692163241242647] Cloyes KG Rosenkranz SJ Supiano KP , et al. Caring to learn and learning to care: inmate hospice volunteers and the delivery of prison end-of-life care. J Correct Health Care 2017; 23(1): 43–55. 28100141 10.1177/1078345816684833 PMC5484572

[bibr37-02692163241242647] Depner RM Grant PC Byrwa DJ , et al. “People don’t understand what goes on in here”: a consensual qualitative research analysis of inmate-caregiver perspectives on prison-based end-of-life care. Palliat Med 2018; 32(5): 969–979. 29432706 10.1177/0269216318755624

[bibr38-02692163241242647] Loeb SJ Hollenbeak CS Penrod J , et al. Care and companionship in an isolating environment: inmates attending to dying peers. J Forensic Nurs 2013; 9(1): 7. 10.1097/JFN.0b013e31827a585c PMC3809039 24158099

[bibr39-02692163241242647] Wright KN Bronstein L. An organizational analysis of prison hospice. Prison J 2007; 87(4): 391–407.

[40] Yampolskaya S Winston N. Hospice care in prison: general principles and outcomes. Am J Hosp Palliat Care 2003; 20(4): 290–296. 12911074 10.1177/104990910302000411

[41] Turner M Peacock M. Palliative care in UK prisons: practical and emotional challenges for staff and fellow prisoners. J Correct Health Care 2017; 23(1): 56–65. 28100148 10.1177/1078345816684847

[bibr42-02692163241242647] Turner M Payne S Barbarachild Z. Care or custody? An evaluation of palliative care in prisons in North West England. Palliat Med 2011; 25(4): 4. 10.1177/0269216310393058 21239467

[43] Turner M Peacock M Payne S , et al. Ageing and dying in the contemporary neoliberal prison system: exploring the ‘double burden’ for older prisoners. Soc Sci Med 2018; 212: 161–167. 10.1016/j.socscimed.2018.07.009 30031982

[44] McParland C Johnston B. Caring, sharing, preparing and declaring: how do hospices support prisons to provide palliative and end of life care? A qualitative descriptive study using telephone interviews. Palliat Med 2021; 35(3): 563–573. 33302784 10.1177/0269216320979194 PMC7975864

[45] Shaw J Driftmier P. “Dying with a smile, just knowing that somebody’s listened to me”: end-of-life care and medical assistance in dying in Canadian prisons. Omega 2024; 88: 1290–1313. 34971334 10.1177/00302228211052341 PMC10848607

[46] Chassagne A Godard A Cretin E , et al. The collision of inmate and patient: end-of-life issues in french prisons. J Correct Health Care 2017; 23(1): 66–75. 28100139 10.1177/1078345816685084

[47] Panozzo S Bryan T Collins A , et al. Complexities and constraints in end-of-life care for hospitalized prisoner patients. J Pain Symptom Manage. 2020; 60(5): 984–991.e1. 10.1016/j.jpainsymman.2020.05.024 32464261

[48] Humane Prison Hospice Model. https://hospicecare.com/uploads/2022/2/Humane-Prison-Hospice-Project-Model.pdf (2022).

[bibr49-02692163241242647] Inside the prison hospice where no inmate dies alone. Reuters. https://www.reuters.com/article/us-california-aging-hospice/inside-the-prison-hospice-where-no-inmate-dies-alone-idUSKBN1JF1XC (2018, accessed 20 June 2023)

[bibr50-02692163241242647] An inmate serving 50 years for attempted murder is an unlikely caretaker for dying prisoners. https://www.bangordailynews.com/2020/02/08/news/midcoast/an-inmate-serving-50-years-for-attempted-murder-is-an-unlikely-caretaker-for-dying-prisoners/ (2020, accessed 20 June 2023)

[51] Jaouad S. The prisoners who care for the dying and get another chance at life. The New York Times. https://www.nytimes.com/interactive/2018/05/16/magazine/health-issue-convicted-prisoners-becoming-caregivers.html?mtrref=undefined&gwh=52CAA3210F4C2542348BF8BDC78E748C&gwt=pay&assetType=PAYWALL (2018, accessed 20 June 2023)

[52] Peacock M Turner M Varey S. “We call it jail craft”: the erosion of the protective discourses drawn on by prison officers dealing with ageing and dying prisoners in the neoliberal, carceral system. Sociology 2018; 52(6): 1152–1168. 30587877 10.1177/0038038517695060 PMC6287248

[53] Australian Government Department of Health. Exploratory analysis of barriers to palliative care. Issues report on people who are incarcerated. https://www.ahaconsulting.com.au (2019, accessed 22 October 2021.)

[54] Agenda: National Conference on Correctional Health Care 2022. https://events.ncchc.org/national-conference/agenda (2023, accessed 10 May 2023).

[55] Kemp R Milton L Michie G , et al. P-43 Palliative care for prisoners: a partnership approach. BMJ Support Palliat Care 2018; 8(Suppl 2): A25–A26.

[56] Fletcher A Payne S Waterman D , et al. Palliative and end of life care in prisons in england and wales – approaches taken to improve inequalities. BMJ Support Palliat Care 2014; 4(Suppl 1): A19.

[57] Norwich. The Good Book of Prisons. https://www.goodbookofprisons.com/norwich/ (2019, accessed 5 July 2023).

[58] Delivering end of life care in prison. Hospice UK. https://www.hospiceuk.org/latest-from-hospice-uk/delivering-end-life-care-prison (2019, accessed 5 July 2023).

[59] California can find better ways of dealing with dying prisoners. https://eu.desertsun.com/story/opinion/2022/06/22/california-can-find-better-ways-dealing-dying-prisoners/7702791001/ (accessed 20 June 2023).

[bibr60-02692163241242647] Compassionate release for aging prisoners isn’t enough. Cognoscenti. https://www.wbur.org/cognoscenti/2022/05/24/compassionate-release-armand-coleman-sarah-laughlin (2022, accessed 20 June 2023).

[bibr61-02692163241242647] Dying in dignity behind bars. SWI swissinfo.ch. https://www.swissinfo.ch/eng/elderly-prisoners_dying-in-dignity-behind-bars/45178650 (2019, accessed 26 June 2023).

[62] Terminally-ill inmate Vicki Letele talks aboutfitness familyandalternative medicineNZ Herald. https://www.nzherald.co.nz/nz/terminally-ill-inmate-vicki-letele-talks-about-fitness-family-and-alternative-medicine/AGGST-6Q5UCFTG2MLDEXCQSH4DM/ (accessed 5 July 2023).

[63] Investigation Report into the circumstances surrounding the death of Mr I 2018. Inspector of Prisons. https://www.oip.ie/investigation-report-into-the-circumstances-surrounding-the-death-of-mr-i-2018/ (2020, accessed 12 August 2022).

[64] Independent Advisory Panel on Deaths in Custody and RCN. Avoidable natural deaths in prison custody: putting things right (September 2020). Prison setting. Patient Safety Learning – the hub. https://www.pslhub.org/learn/patient-safety-in-health-and-care/care-settings/prison-setting/independent-advisory-panel-on-deaths-in-custody-and-rcn-avoidable-natural-deaths-in-prison-custody-putting-things-right-september-2020-r3263/ (2020, accessed 27 June 2023).

[65] Framework on Palliative Care in Canada. Canada.ca. https://www.canada.ca/en/health-canada/services/health-care-system/reports-publications/palliative-care/framework-palliative-care-canada.html#p2.3.1 (2018, acc-essed 21 February 2023).

[66] Dying Behind Bars: How can we better support people in prison at the end of life? Hospice UK. https://www.hospiceuk.org/publications-and-resources/dying-behind-bars-how-can-we-better-support-people-prison-end-life (2021, accessed 5 July 2023).

[67] NHS England. Dying well in custody charter and self ass-essment tool. https://www.england.nhs.uk/publication/dying-well-in-custody-charter-and-self-assessment-tool/ (2018, accessed 29 August 2023).

