# Supplementary material for: How do people in prison access palliative care? A scoping review of models of palliative care delivery for people in prison in high-income countries
Source: Palliat Med. 2024 Apr 16;38(5):517–34. doi: 10.1177/02692163241242647 (PMC11107131; doi:10.1177/02692163241242647)
Supplement: sj-pdf-1-pmj-10.1177_02692163241242647 – Supplemental material for How do people in prison access palliative care? A scoping review of models of palliative care delivery for people in prison in high-income countries [file sj-pdf-1-pmj-10.1177_02692163241242647.pdf]

Table 4. Qualitative Studies

MMAT Criteria	Brooke Cooley Webb et al.2023	Chassagne et al. 2017 (France)	Cloyes et al.2016 (USA)	Loeb et al. 2013 (USA)	Panozzo et al. 2020 (Australia)	Steely Smith et al. 2022 (USA)	Wright et al. 200 (USA)	Yampolskaya et al. 2003 (USA)	Bronstein (USA) 2007	McParland et al. 2021 (Scotland)	Turner et al. 2017 (UK)	Shaw et al. 2021 (Canada)	Cichowlas et al. 2009 (USA)
1.1 Is the qualitative approach appropriate to answer the research question?	Yes	Yes	Yes	Yes	Yes	Yes	Yes	Yes	Yes	Yes	Yes	Can't Tell	Can't Tell
1.2 Are the qualitative data collection methods adequate to address the research question?	Yes	Yes	Yes	Yes	Yes	Yes	Yes	Yes	Can't Tell	Can't Tell	Yes	Yes	Can't Tell
1.3. Are the findings adequately derived from the data?	Yes	Yes	Yes	Yes	Yes	Yes	Yes	Yes	Yes	Yes	Can't Tell	Yes	Can't Tell
1.4. Is the interpretation of results sufficiently substantiated by data?	Yes	Yes	Yes	Yes	Yes	Yes	Yes	Yes	Yes	Yes	Yes	Yes	Can't Tell

1.5. Is there coherence between qualitative data sources, collection, analysis and interpretation?	Yes	Yes	Yes	Yes	Yes	Yes	Yes	Yes	Yes	Yes	Yes	Yes	Can't Tell
Rationale for score	All screening criteria met.	All screening criteria met.	All screening criteria met.	All screening criteria met.	All screening criteria met.	All screening criteria met.	All screening criteria met.	All screening criteria met	Data collection method of telephone surveys not as robust as face to face.	Brief phone interviews limit depth compared to face-to-face interviews. Broader representation from various staff groups is needed for a comprehensive	Study doesn't report on thematic approach utilised by the research team.	There is ambiguity in the research question, and the clarity of integrating data from the document review is lacking.	A lack of clarity in the research question, reliance on a single data collection source with measurement against pre-existing themes, an unrepresentative sample size, and the absence of methodology and analysis discussions.

Table 5. Quantitative Descriptive Studies	
**MMAT Criteria**	**Hoffman et al., 2011 (USA)**
4.1 Is the sampling strategy relevant to address the research question?	Yes
4,2. Is the sample representative of the target population?	Yes
4.3. Are the measurements appropriate?	Yes
4.4. Is the risk of nonresponse bias low?	Yes
4.5 Is the statistical analysis appropriate to answer the research question?	Yes
Rationale for score	All screening criteria met.

Table 6. Mixed Methods Studies						
**MMAT Criteria**	Byock et al. 2006 (USA)	Papadopoulos et al. 2016 (UK)	Prost et al 2020 (USA)	Turner et al. 2011 (UK)	Turner et al. 2018 (UK)	Turner et al. 2021 (Multiple Countries)
5.1. Is there an adequate rationale for using a mixed methods design to address the research question?	Yes	Yes	No	Yes	Yes	Yes
5.2. Are the different components of the study effectively integrated to answer the research question?	Yes	Can't Tell	Can't Tell	Yes	Yes	Yes
5.3. Are the outputs of the integration of qualitative and quantitative components adequately interpreted?	Yes	Can't Tell	No	Yes	Yes	Yes

5.4. Are divergences and inconsistencies between quantitative and qualitative results adequately addressed?	Yes	Yes	No	Yes	Yes	Yes
5.5. Do the different components of the study adhere to the quality criteria of each tradition of the methods involved?	Yes	Can't Tell	Can't Tell	Yes	Yes	Can't Tell
Rationale for score	All screening criteria met.	Themes lacked support from verbatim quotes, limiting the meta-inference. Additionally, the qualitative component was insufficiently reported, lacking details on coding and theme development.	Ineffective integration of study components to address the research question. The handling of free text data, interpretation of outputs from integrating qualitative and quantitative components, and addressing divergences and inconsistencies were inadequately discussed.	All screening criteria met.	All screening criteria met.	No detail about thematic approach used to analyse the free text data.

Supplementary Material 1. Social sciences citation index web of science search strategy

#9 AND

#10 and **2000** or **2001** or **2002** or **2003** or **2004** or **2005** or **2022** or **2021** or **2020** or **2019** or **2018** or **2017** or **2016** or **2015** or **2014** or **2013** or **2012** or **2011** or **2010** or **2009** or **2008** or **2007** or **2006** (Publication Years)

Prion* OR Prisoner* OR Jail* OR Incarcerat* OR "Correctional Facilities" OR Convict* OR felon* OR offender* OR inmate* OR penitentiar* OR gaol (Topic)

"Palliative Care" OR "Terminal Care" OR Death OR '"Terminally ill" OR "Hospice Care" OR "Pain Management" OR "Advance Directive*"OR "Advance Care Planning" OR Palliat* OR "end-of-life" OR "terminal illness" OR dying OR "end stage illness" OR "supportive care" OR "symptom management" OR "compassionate release" (Topic)

Supplementary Material 2. Psyinfo search strategy

1.	Palliative care.mp. or exp palliative therapy/
2.	Terminal Care.mp. or exp terminal care/
3.	exp death/ or Death.mp.
4.	Terminally ill.mp. or exp terminally ill patient/
5.	Hospice Care.mp. or exp hospice care/
6.	'Pain management'.mp.
7.	'Advance* Directive*'.mp.
8.	'Advance* Care Planning'.mp. or exp advance care planning/
9.	Palliat*.mp.
10.	'end of life'.mp.
11.	'end of life care'.mp.
12.	'terminal illness'.mp. or exp terminal disease/
13.	dying.mp. or exp dying/
14.	'end stage illness'.mp.
15.	'supportive care'.mp.
16.	'symptom management'.mp.
17.	'compassionate release'.mp.
18.	1 or 2 or 3 or 4 or 5 or 6 or 7 or 8 or 9 or 10 or 11 or 12 or 13 or 14 or 15 or 16 or 17
19.	Prison*.mp. or exp prison/ or exp prison nursing/ or exp prisoner/

20.	Criminal*.mp. or exp criminal justice/ or exp criminal behavior/
21.	Jail*.mp.
22.	exp incarceration/ or Incarcerat*.mp.
23.	'Correctional Facilities'.mp. or exp correctional facility/
24.	convict*.mp.
25.	felon*.mp.
26.	exp offender/ or offender*.mp.
27.	inmate*.mp.
28.	penitentiar*.mp.
29.	gaol*.mp.
30.	secure.mp.
31.	19 or 20 or 21 or 22 or 23 or 24 or 25 or 26 or 27 or 28 or 29 or 30
32.	18 and 31

Supplementary Material 3. CINAHL search strategy

S57	(S41 OR S42 OR S43 OR S44 OR S45 OR S46 OR S47 OR S48 OR S49 OR S50 OR S51 OR S52 OR S53)) AND (S40 AND S54)	Limiters - Published Date: 20000101-20221231<br>Expanders - Apply equivalent subjects<br>Search modes - Boolean/Phrase	Interface - EBSCOhost Research Databases<br>Search Screen - Advanced Search<br>Database - CINAHL	1,394
S56	(S41 OR S42 OR S43 OR S44 OR S45 OR S46 OR S47 OR S48 OR S49 OR S50 OR S51 OR S52 OR S53)) AND (S40 AND S54)	Limiters - Published Date: 20000101-20221231<br>Expanders - Apply equivalent subjects<br>Search modes - Boolean/Phrase	Interface - EBSCOhost Research Databases<br>Search Screen - Advanced Search<br>Database - CINAHL	1,394
S55	(S41 OR S42 OR S43 OR S44 OR S45 OR S46 OR S47 OR S48 OR S49 OR S50 OR S51 OR S52 OR S53)) AND (S40 AND S54)	Expanders - Apply equivalent subjects<br>Search modes - Boolean/Phrase	Interface - EBSCOhost Research Databases<br>Search Screen - Advanced Search<br>Database - CINAHL	Display
S54	S41 OR S42 OR S43 OR S44 OR S45 OR S46 OR S47 OR S48 OR S49 OR S50 OR S51 OR S52 OR S53)	Expanders - Apply equivalent subjects<br>Search modes - Boolean/Phrase	Interface - EBSCOhost Research Databases<br>Search Screen - Advanced Search<br>Database - CINAHL	Display
S53	"secure"	Expanders - Apply equivalent subjects<br>Search modes - Boolean/Phrase	Interface - EBSCOhost Research Databases	Display

			Search Screen - Advanced Search<br>Database - CINAHL	
S52	"gaol"	Expanders - Apply equivalent subjects<br>Search modes - Boolean/Phrase	Interface - EBSCOhost Research Databases<br>Search Screen - Advanced Search<br>Database - CINAHL	Display
S51	"penitentiar*"	Expanders - Apply equivalent subjects<br>Search modes - Boolean/Phrase	Interface - EBSCOhost Research Databases<br>Search Screen - Advanced Search<br>Database - CINAHL	Display
S50	"inmate*"	Expanders - Apply equivalent subjects<br>Search modes - Boolean/Phrase	Interface - EBSCOhost Research Databases<br>Search Screen - Advanced Search<br>Database - CINAHL	Display
S49	"offender*"	Expanders - Apply equivalent subjects<br>Search modes - Boolean/Phrase	Interface - EBSCOhost Research Databases<br>Search Screen - Advanced Search<br>Database - CINAHL	Display

S48	"felon*"	Expanders - Apply equivalent subjects<br>Search modes - Boolean/Phrase	Interface - EBSCOhost Research Databases<br>Search Screen - Advanced Search<br>Database - CINAHL	Display
S47	"convict*"	Expanders - Apply equivalent subjects<br>Search modes - Boolean/Phrase	Interface - EBSCOhost Research Databases<br>Search Screen - Advanced Search<br>Database - CINAHL	Display
S46	(MH "Correctional Facilities")	Expanders - Apply equivalent subjects<br>Search modes - Boolean/Phrase	Interface - EBSCOhost Research Databases<br>Search Screen - Advanced Search<br>Database - CINAHL	Display
S45	"Incarcerat*"	Expanders - Apply equivalent subjects<br>Search modes - Boolean/Phrase	Interface - EBSCOhost Research Databases<br>Search Screen - Advanced Search<br>Database - CINAHL	Display
S44	"Jail*"	Expanders - Apply equivalent subjects<br>Search modes - Boolean/Phrase	Interface - EBSCOhost Research Databases	Display

			Search Screen - Advanced Search<br>Database - CINAHL	
S43	"Criminal*"	Expanders - Apply equivalent subjects<br>Search modes - Boolean/Phrase	Interface - EBSCOhost Research Databases<br>Search Screen - Advanced Search<br>Database - CINAHL	Display
S42	(MH "Prisoners")	Expanders - Apply equivalent subjects<br>Search modes - Boolean/Phrase	Interface - EBSCOhost Research Databases<br>Search Screen - Advanced Search<br>Database - CINAHL	Display
S41	"Prison*"	Expanders - Apply equivalent subjects<br>Search modes - Boolean/Phrase	Interface - EBSCOhost Research Databases<br>Search Screen - Advanced Search<br>Database - CINAHL	Display
S40	S21 OR S22 OR S23 OR S24 OR S25 OR S26 OR S27 OR S28 OR S29 OR S30 OR S31 OR S32 OR S33 OR S34 OR S35 OR S36 OR S37 OR S38 OR S39	Expanders - Apply equivalent subjects<br>Search modes - Boolean/Phrase	Interface - EBSCOhost Research Databases<br>Search Screen - Advanced Search<br>Database - CINAHL	177,157

S39	"'compassionate release'"	Expanders - Apply equivalent subjects<br>Search modes - Boolean/Phrase	Interface - EBSCOhost Research Databases<br>Search Screen - Advanced Search<br>Database - CINAHL	Display
S38	"'symptom management'"	Expanders - Apply equivalent subjects<br>Search modes - Boolean/Phrase	Interface - EBSCOhost Research Databases<br>Search Screen - Advanced Search<br>Database - CINAHL	Display
S37	"'supportive care'"	Expanders - Apply equivalent subjects<br>Search modes - Boolean/Phrase	Interface - EBSCOhost Research Databases<br>Search Screen - Advanced Search<br>Database - CINAHL	Display
S36	"'end stage illness'"	Expanders - Apply equivalent subjects<br>Search modes - Boolean/Phrase	Interface - EBSCOhost Research Databases<br>Search Screen - Advanced Search<br>Database - CINAHL	Display
S35	"dying"	Expanders - Apply equivalent subjects<br>Search modes - Boolean/Phrase	Interface - EBSCOhost Research Databases	Display

			Search Screen - Advanced Search<br>Database - CINAHL	
S34	dying	Expanders - Apply equivalent subjects<br>Search modes - Boolean/Phrase	Interface - EBSCOhost Research Databases<br>Search Screen - Advanced Search<br>Database - CINAHL	Display
S33	"terminal illness"	Expanders - Apply equivalent subjects<br>Search modes - Boolean/Phrase	Interface - EBSCOhost Research Databases<br>Search Screen - Advanced Search<br>Database - CINAHL	Display
S32	'"end-of-life care"'	Expanders - Apply equivalent subjects<br>Search modes - Boolean/Phrase	Interface - EBSCOhost Research Databases<br>Search Screen - Advanced Search<br>Database - CINAHL	Display
S31	'"end-of-life"'	Expanders - Apply equivalent subjects<br>Search modes - Boolean/Phrase	Interface - EBSCOhost Research Databases<br>Search Screen - Advanced Search<br>Database - CINAHL	Display

S30	"Palliat*"	Expanders - Apply equivalent subjects<br>Search modes - Boolean/Phrase	Interface - EBSCOhost Research Databases<br>Search Screen - Advanced Search<br>Database - CINAHL	Display
S29	(MH "Advance Care Planning")	Expanders - Apply equivalent subjects<br>Search modes - Boolean/Phrase	Interface - EBSCOhost Research Databases<br>Search Screen - Advanced Search<br>Database - CINAHL	Display
S28	(MH "Advance Directives+")	Expanders - Apply equivalent subjects<br>Search modes - Boolean/Phrase	Interface - EBSCOhost Research Databases<br>Search Screen - Advanced Search<br>Database - CINAHL	Display
S27	(MH "Pain Management")	Expanders - Apply equivalent subjects<br>Search modes - Boolean/Phrase	Interface - EBSCOhost Research Databases<br>Search Screen - Advanced Search<br>Database - CINAHL	Display
S26	(MH "Hospice Care")	Expanders - Apply equivalent subjects<br>Search modes - Boolean/Phrase	Interface - EBSCOhost Research Databases	Display

			Search Screen - Advanced Search<br>Database - CINAHL	
S25	Terminally ill	Expanders - Apply equivalent subjects<br>Search modes - Boolean/Phrase	Interface - EBSCOhost Research Databases<br>Search Screen - Advanced Search<br>Database - CINAHL	Display
S24	(MH "Terminally Ill Patients+")	Expanders - Apply equivalent subjects<br>Search modes - Boolean/Phrase	Interface - EBSCOhost Research Databases<br>Search Screen - Advanced Search<br>Database - CINAHL	Display
S23	(MH "Death+")	Expanders - Apply equivalent subjects<br>Search modes - Boolean/Phrase	Interface - EBSCOhost Research Databases<br>Search Screen - Advanced Search<br>Database - CINAHL	Display
S22	(MH "Terminal Care+")	Expanders - Apply equivalent subjects<br>Search modes - Boolean/Phrase	Interface - EBSCOhost Research Databases<br>Search Screen - Advanced Search<br>Database - CINAHL	Display

S21	(MH "Palliative Care")	Expanders - Apply equivalent subjects<br>Search modes - Boolean/Phrase	Interface - EBSCOhost Research Databases<br>Search Screen - Advanced Search<br>Database - CINAHL	Display
S20	((AB dying OR AB 'end stage illness' OR AB 'supportive care' OR AB 'symptom management' OR AB 'compassionate release') AND (S17 AND S18)) AND (S16 AND S19)	Expanders - Apply equivalent subjects<br>Search modes - Boolean/Phrase	Interface - EBSCOhost Research Databases<br>Search Screen - Advanced Search<br>Database - CINAHL	149
S19	(AB dying OR AB 'end stage illness' OR AB 'supportive care' OR AB 'symptom management' OR AB 'compassionate release') AND (S17 AND S18)	Expanders - Apply equivalent subjects<br>Search modes - Boolean/Phrase	Interface - EBSCOhost Research Databases<br>Search Screen - Advanced Search<br>Database - CINAHL	13,174
S18	AB dying OR AB 'end stage illness' OR AB 'supportive care' OR AB 'symptom management' OR AB 'compassionate release'	Expanders - Apply equivalent subjects<br>Search modes - Boolean/Phrase	Interface - EBSCOhost Research Databases<br>Search Screen - Advanced Search<br>Database - CINAHL	29,549

S17	AB palliative care OR AB terminal care OR AB death OR AB terminally ill OR AB hospice care OR AB 'pain management' OR AB advance* directives OR AB advance care planning OR AB Palliat* OR AB end-of-life OR AB 'end of life care' OR AB terminal illness	Expanders - Apply equivalent subjects<br>Search modes - Boolean/Phrase	Interface - EBSCOhost Research Databases<br>Search Screen - Advanced Search<br>Database - CINAHL	235,860
S16	AB ((MH "Prisoners") OR (MH "Correctional Facilities") OR (MH "Correctional Health Services")) OR AB criminal* OR AB jail* OR AB convict* OR AB felon* OR AB offender* OR AB inmate* OR AB penitentiar* OR AB incarcerat* OR AB gaol* OR AB secure OR AB prison*	Expanders - Apply equivalent subjects<br>Search modes - Boolean/Phrase	Interface - EBSCOhost Research Databases<br>Search Screen - Advanced Search<br>Database - CINAHL	40,659
S15	((AB dying OR AB 'end stage illness' OR AB 'supportive care' OR AB 'symptom management' OR AB	Expanders - Apply equivalent subjects<br>Search modes - Boolean/Phrase	Interface - EBSCOhost Research Databases	149

	'compassionate release') AND (S12 AND S13)) AND (S11 AND S14)		Search Screen - Advanced Search<br>Database - CINAHL	
S14	(AB dying OR AB 'end stage illness' OR AB 'supportive care' OR AB 'symptom management' OR AB 'compassionate release') AND (S12 AND S13)	Expanders - Apply equivalent subjects<br>Search modes - Boolean/Phrase	Interface - EBSCOhost Research Databases<br>Search Screen - Advanced Search<br>Database - CINAHL	13,174
S13	AB dying OR AB 'end stage illness' OR AB 'supportive care' OR AB 'symptom management' OR AB 'compassionate release'	Expanders - Apply equivalent subjects<br>Search modes - Boolean/Phrase	Interface - EBSCOhost Research Databases<br>Search Screen - Advanced Search<br>Database - CINAHL	29,549
S12	AB palliative care OR AB terminal care OR AB death OR AB terminally ill OR AB hospice care OR AB 'pain management' OR AB advance* directives OR AB advance care planning OR AB Palliat* OR AB end-of-life OR AB 'end of life care' OR AB terminal illness	Expanders - Apply equivalent subjects<br>Search modes - Boolean/Phrase	Interface - EBSCOhost Research Databases<br>Search Screen - Advanced Search<br>Database - CINAHL	235,860

S11	AB ((MH "Prisoners") OR (MH "Correctional Facilities") OR (MH "Correctional Health Services")) OR AB criminal* OR AB jail* OR AB convict* OR AB felon* OR AB offender* OR AB inmate* OR AB penitentiar* OR AB incarcerat* OR AB gaol* OR AB secure OR AB prison*	Expanders - Apply equivalent subjects<br>Search modes - Boolean/Phrase	Interface - EBSCOhost Research Databases<br>Search Screen - Advanced Search<br>Database - CINAHL	40,659
S10	((AB dying OR AB 'end stage illness' OR AB 'supportive care' OR AB 'symptom management' OR AB 'compassionate release') AND (S7 AND S8)) AND (S6 AND S9)	Expanders - Apply equivalent subjects<br>Search modes - Boolean/Phrase	Interface - EBSCOhost Research Databases<br>Search Screen - Advanced Search<br>Database - CINAHL	149
S9	(AB dying OR AB 'end stage illness' OR AB 'supportive care' OR AB 'symptom management' OR AB 'compassionate release') AND (S7 AND S8)	Expanders - Apply equivalent subjects<br>Search modes - Boolean/Phrase	Interface - EBSCOhost Research Databases<br>Search Screen - Advanced Search<br>Database - CINAHL	13,174

S8	AB dying OR AB 'end stage illness' OR AB 'supportive care' OR AB 'symptom management' OR AB 'compassionate release'	Expanders - Apply equivalent subjects<br>Search modes - Boolean/Phrase	Interface - EBSCOhost Research Databases<br>Search Screen - Advanced Search<br>Database - CINAHL	29,549
S7	AB palliative care OR AB terminal care OR AB death OR AB terminally ill OR AB hospice care OR AB 'pain management' OR AB advance* directives OR AB advance care planning OR AB Palliat* OR AB end-of-life OR AB 'end of life care' OR AB terminal illness	Expanders - Apply equivalent subjects<br>Search modes - Boolean/Phrase	Interface - EBSCOhost Research Databases<br>Search Screen - Advanced Search<br>Database - CINAHL	235,860
S6	AB ((MH "Prisoners") OR (MH "Correctional Facilities") OR (MH "Correctional Health Services")) OR AB criminal* OR AB jail* OR AB convict* OR AB felon* OR AB offender* OR AB inmate* OR AB penitentiar* OR AB incarcerat* OR	Expanders - Apply equivalent subjects<br>Search modes - Boolean/Phrase	Interface - EBSCOhost Research Databases<br>Search Screen - Advanced Search<br>Database - CINAHL	40,659

	AB gaol* OR AB secure OR AB prison*			
S5	((AB dying OR AB 'end stage illness' OR AB 'supportive care' OR AB 'symptom management' OR AB 'compassionate release') AND (S2 AND S3)) AND (S1 AND S4)	Expanders - Apply equivalent subjects<br>Search modes - Boolean/Phrase	Interface - EBSCOhost Research Databases<br>Search Screen - Advanced Search<br>Database - CINAHL	149
S4	(AB dying OR AB 'end stage illness' OR AB 'supportive care' OR AB 'symptom management' OR AB 'compassionate release') AND (S2 AND S3)	Expanders - Apply equivalent subjects<br>Search modes - Boolean/Phrase	Interface - EBSCOhost Research Databases<br>Search Screen - Advanced Search<br>Database - CINAHL	13,174
S3	AB dying OR AB 'end stage illness' OR AB 'supportive care' OR AB 'symptom management' OR AB 'compassionate release'	Expanders - Apply equivalent subjects<br>Search modes - Boolean/Phrase	Interface - EBSCOhost Research Databases<br>Search Screen - Advanced Search<br>Database - CINAHL	29,549
S2	AB palliative care OR AB terminal care OR AB death OR AB terminally ill OR AB hospice care OR AB 'pain	Expanders - Apply equivalent subjects<br>Search modes - Boolean/Phrase	Interface - EBSCOhost Research Databases	235,860

	management' OR AB advance* directives OR AB advance care planning OR AB Palliat* OR AB end-of-life OR AB 'end of life care' OR AB terminal illness		Search Screen - Advanced Search<br>Database - CINAHL	
S1	AB ((MH "Prisoners") OR (MH "Correctional Facilities") OR (MH "Correctional Health Services")) OR AB criminal* OR AB jail* OR AB convict* OR AB felon* OR AB offender* OR AB inmate* OR AB penitentiar* OR AB incarcerat* OR AB gaol* OR AB secure OR AB prison*	Expanders - Apply equivalent subjects<br>Search modes - Boolean/Phrase	Interface - EBSCOhost Research Databases<br>Search Screen - Advanced Search<br>Database - CINAHL	40,659

Supplementary Material 4. PSYCHINFO final search

1.	Palliative care.mp. or exp palliative therapy/
2.	Terminal Care.mp. or exp terminal care/
3.	exp death/ or Death.mp.
4.	Terminally ill.mp. or exp terminally ill patient/
5.	Hospice Care.mp. or exp hospice care/
6.	'Pain management'.mp.
7.	'Advance* Directive*'.mp.
8.	'Advance* Care Planning'.mp. or exp advance care planning/
9.	Palliat*.mp.
10.	'end of life'.mp.
11.	'end of life care'.mp.
12.	'terminal illness'.mp. or exp terminal disease/
13.	dying.mp. or exp dying/
14.	'end stage illness'.mp.
15.	'supportive care'.mp.
16.	'symptom management'.mp.
17.	'compassionate release'.mp.
18.	1 or 2 or 3 or 4 or 5 or 6 or 7 or 8 or 9 or 10 or 11 or 12 or 13 or 14 or 15 or 16 or 17

19.	Prison*.mp. or exp prison/ or exp prison nursing/ or exp prisoner/				
20.	Criminal*.mp. or exp criminal justice/ or exp criminal behavior/				
21.	Jail*.mp.				
22.	exp incarceration/ or Incarcerat*.mp.				
23.	'Correctional Facilities'.mp. or exp correctional facility/				
24.	convict*.mp.				
25.	felon*.mp.				
26.	exp offender/ or offender*.mp.				
27.	inmate*.mp.				
28.	penitentiar*.mp.				
29.	gaol*.mp.				
30.	secure.mp.				
31.	19 or 20 or 21 or 22 or 23 or 24 or 25 or 26 or 27 or 28 or 29 or 30				
32.	18 and 31				

Supplementary Material 5. EMBASE search strategy

#	Query	Results from 10 Dec 2021
1	exp palliative therapy/	123,114
2	exp terminal care/	75,351
3	exp death/	759,351
4	exp terminally ill patient/	9,036
5	exp hospice care/	11,520
6	"pain management".mp.	43,759
7	'advance directives'.mp.	4,797
8	exp advance care planning/	4,222
9	Palliat*.mp.	175,448
10	"end-of-life".mp.	40,184
11	"end-of-life care".mp.	17,734
12	exp terminal disease/	6,724
13	"terminal illness".mp.	2,803
14	exp dying/	9,567
15	"end stage illness".mp.	40
16	"supportive care".mp.	34,503

17	"symptom management".mp.	10,031
18	"compassionate release".mp.	49
19	Palliative care.mp.	56,754
20	Terminal care.mp.	39,269
21	death.mp.	1,391,485
22	terminally ill.mp.	12,347
23	hospice care.mp.	13,167
24	exp hospice/	14,572
25	advance care planning.mp. or advance care planning/	6,901
26	dying.mp.	52,644
27	prisoner/ or prison/	28,044
28	prison.mp.	21,019
29	prisoner.mp.	18,532
30	Criminal*.mp.	40,633
31	Jail*.mp.	4,756
32	Incarcerat*.mp.	16,675
33	correctional facility/	1,068
34	exp correctional facility/	1,391
35	convict*.mp.	9,041

36	felon*.mp.	996
37	exp offender/	14,938
38	offender.mp.	18,336
39	inmate*.mp.	6,415
40	penitentiar*.mp.	848
41	gaol*.mp.	179
42	1 or 2 or 3 or 4 or 5 or 6 or 7 or 8 or 9 or 10 or 11 or 12 or 13 or 14 or 15 or 16 or 17 or 18 or 19 or 20 or 21 or 22 or 23 or 24 or 25 or 26	1,866,709
43	27 or 28 or 29 or 30 or 31 or 32 or 33 or 34 or 35 or 36 or 37 or 38 or 39 or 40 or 41	95,613
44	42 and 43	9,216
45	limit 44 to yr="2000 -Current"	6,992

Supplementary Material 6. Data Extraction Template

Title
Type of study
Lead author info
Prison category or type
Country
Participants (details e.g. age/sex and number)

Who delivers the care (volunteer, prison nurse, specialist in-reach professional etc, interdisciplinary team etc)
Where does the care take place?
How are social care needs considered?
how are psychological needs considered? (needs essential to mental health/ not a biological necessity)
how are spiritual needs considered?
What is the admission criteria for hospice or palliative care?
What opportunities are there for multi-disciplinary and partnership working? Who are the partners?
Is there any evidence of specialist provision for older prisoners?
Can you describe the intervention/interventions delivered in this model? Has any consideration been given to the need for specialist palliative care (ie care that can't be delivered by the current care team)?
What prison policies are in place to support the care – eg visitation policies, Release On Temporary Licence and Early Release on Compassionate Grounds discussed?
Are the rights of prisoners' families or loved ones taken into consideration?
Is bereavement support offered to fellow prisoners, loved ones or prison staff?
What are the outcomes and benefits to prisoners or stakeholders of this model?
What are the facilitators and challenges of delivering this model?

What are the funding implications of delivering EOL care in the prison setting? Is it discussed?

Table 2. MEDLINE search strategy

MEDLINE Search Strategy	
1	Palliative Care/
2	Terminal Care/
3	Death/
4	Terminally Ill/
5	Hospice Care/
6	Pain Management/
7	Advance Directives/
8	Advance Care Planning/
10	Palliat*.mp.
11	end of life.mp.
12	end of life care.mp.
13	terminal illness.mp.
14	dying.mp.

15	end stage illness.mp.
16	supportive care.mp.
17	symptom management.mp.
18	compassionate release.mp.
19	1 or 2 or 3 or 4 or 5 or 6 or 7 or 8 or 9 or 10 or 11 or 12 or 13 or 14 or 15 or 16 or 17 or 18
20	People in prison/ or Prisons/
21	Criminals/
22	Jails/
23	Incarcerat*mp.
23	Correctional Facilities/
24	convict*.mp.
25	felon*.mp.
26	offender*.mp.
27	inmate*.mp.
28	penitentiar*.mp. [
29	gaol.mp.
30	secure.mp.
31	20 or 21 or 22 or 23 or 24 or 25 or 26 or 27 or 28 or 29 or 30

32	19 and 31
